# Plant-water sensitivity regulates wildfire vulnerability

**DOI:** 10.1038/s41559-021-01654-2

**Published:** 2022-02-07

**Authors:** Krishna Rao, A. Park Williams, Noah S. Diffenbaugh, Marta Yebra, Alexandra G. Konings

**Affiliations:** 1 Department of Earth System Science, Stanford University, Stanford, CA USA; 2 Department of Geography, University of California, Los Angeles, CA USA; 3 Lamont–Doherty Earth Observatory, Columbia University, Palisades, NY USA; 4 Woods Institute for the Environment, Stanford University, Stanford, CA USA; 5 Fenner School of Environment & Society, The Australian National University, Acton, Australian Capital Territory Australia; 6 School of Engineering, The Australian National University, Acton, Australian Capital Territory Australia

**Keywords:** Fire ecology, Ecophysiology, Natural hazards, Plant ecology

## Abstract

Extreme wildfires extensively impact human health and the environment. Increasing vapour pressure deficit (VPD) has led to a chronic increase in wildfire area in the western United States, yet some regions have been more affected than others. Here we show that for the same increase in VPD, burned area increases more in regions where vegetation moisture shows greater sensitivity to water limitation (plant-water sensitivity; *R*^2^ = 0.71). This has led to rapid increases in human exposure to wildfire risk, both because the population living in areas with high plant-water sensitivity grew 50% faster during 1990–2010 than in other wildland–urban interfaces and because VPD has risen most rapidly in these vulnerable areas. As plant-water sensitivity is strongly linked to wildfire vulnerability, accounting for ecophysiological controls should improve wildfire forecasts. If recent trends in VPD and demographic shifts continue, human wildfire risk will probably continue to increase.

## Abstract (web summary)

The authors show that an ecosystem’s sensitivity to drought, measured as the amount of change in vegetation moisture content for a given change in background moisture, predicts the fire hazard in that location.

## Main

Wildfire-burne﻿d area has increased rapidly in the western United States over the past four decades^1–3^, threatening humans and altering ecosystem function and vegetation growth patterns^4–6^. Several factors have contributed to the rise in burned area in the western US. Historic fire suppression has increased fuel loads in many areas^7^. Human populations have also expanded the wildland–urban interface (WUI)—a zone of transition between wildland and urban environments^8^—where human-caused ignitions are frequent and vegetation is abundant, posing high risk to human lives and structures^9–11^. Moreover, anthropogenic climate change and natural climate variability have combined to substantially increase the atmospheric aridity, which has contributed to a decline in fuel moisture and a resultant rise in burned area^2^. However, the sensitivity of burned area to atmospheric aridity can vary significantly across regions^12–14^. Understanding and predicting fire hazard as hot, dry conditions become more common^15–17^ require accounting for the factors that modulate this sensitivity, including fuel availability and fuel moisture^18,19^.

As atmospheric aridity increases, live fuel moisture content (LFMC, measured as the mass of plant water per unit dry biomass) generally decreases^20,21^. However, the effect of atmospheric aridity on LFMC is regulated by a range of location-specific factors, including, but not limited to, topographic and soil controls on root-zone water availability, root water uptake and plant hydraulic traits that affect transpirational water loss. Specifically, plant hydraulic traits can cause up to 3-fold variation in LFMC, and thus affect fuel flammability^22^. For the same meteorological conditions (including both atmospheric aridity and precipitation), fuel moisture can vary widely, depending on plant species and hydraulic diversity^23–25^. However, a single fire can impact a wide range of species in a community, with a potentially diverse range of plant hydraulic traits within the area burned^26,27^. It is therefore unclear whether – and if so, to what extent – plant and soil hydraulic traits affect the spatial distribution of LFMC and fire hazard at large scales.

We investigate how the plant and soil features influencing LFMC’s response to climate affect fire hazard. We consider the effects of climate on LFMC through a climate-derived moisture balance that considers both precipitation and vapour pressure deficit (VPD, calculated using the wetness of dead foliage and twigs in the litter; see Methods). We refer to the integrated sensitivity of LFMC to climate-derived moisture balance as plant-water sensitivity (PWS). Because of the significant memory in soil moisture^28^, LFMC depends on both current and previous meteorology. To account for this, we calculate PWS as the sum of the slopes of a multiple linear regression between LFMC anomalies and climate-derived moisture balance anomalies, with lags varying from 0 to 150 d in 15 d intervals.

**Extended Data Fig. 1:**
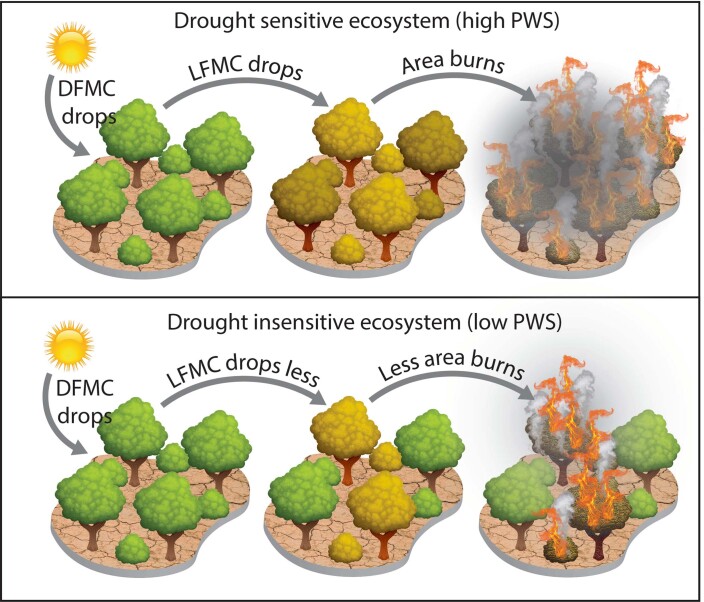
Conceptual diagram showing the effect of plant-water sensitivity (PWS) on burned area. The DFMC denotes dead fuel moisture content. It represents climate-derived moisture balance (see Methods). The LFMC denotes live fuel moisture content.

We hypothesize that PWS regulates the effect of climate on burned area. Specifically, a unit decrease in climate-derived moisture balance will cause a larger decrease in LFMC in ecosystems where PWS is high. This decrease in LFMC may result in higher flammability^29^ and, eventually, a larger burned area than in ecosystems with low PWS where the decrease in LFMC for the same decrease in climate-derived moisture balance is smaller (Extended Data Fig. 1). To test this hypothesis, we use LFMC maps derived from microwave remote sensing^30^. Furthermore, to assess whether current vegetation distributions will buffer or exacerbate future fire vulnerability in the western US, we test the interactions between PWS and other drivers of fire risk, including the rate of change in atmospheric aridity across the region, and the rate of growth of the WUI population.

## Results

### Link between PWS and wildfire vulnerability

**Fig. 1:**
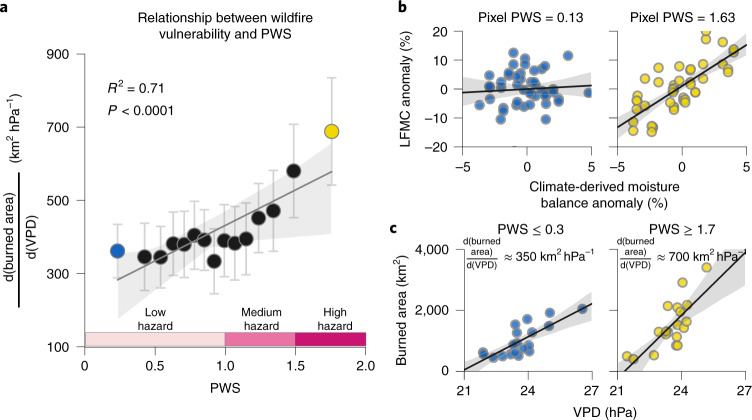
PWS and its link to wildfire vulnerability. **a**, Sensitivity of burned area to VPD as a function of PWS. Points indicate data for 15 equal-vegetated area bins of PWS (Supplementary Fig. 1). Whiskers indicate 1 standard error in the estimate of slope. Pink bars at the bottom represent wildfire hazard due to PWS. **b**, The PWS calculation is illustrated for a sample pixel originating from the first (last) PWS bin and shown on the left (right). In each case, for visual simplicity, only data for the time lag leading to the highest slope between LFMC anomaly and climate-derived moisture balance anomaly are shown, even though PWS is calculated as the sum of all slopes (see Methods). **c**, Annual burned area versus mean annual VPD (April–March) for all pixels in the first (last) PWS bin is shown on the left (right). In all panels, the first (last) PWS bin is indicated by blue (yellow); thick lines indicate linear regression best fits, with grey bands indicating 95% confidence intervals. For the geographic locations of data presented in **b** and **c**, see Supplementary Fig. 2.

The slope between timeseries of annual burned area and annual VPD $\left( {\frac{{{\mathrm{d(burned\;area)}}}}{{{\mathrm{d(VPD)}}}}} \right)$ is strongly linked to the PWS (*R*^2^ = 0.71 and *P* < 0.0001; Fig. 1a). For different bins of PWS, spatially disparate locations with similar PWS are combined and the interannual variations of burned area and VPD is calculated across these locations (Fig. 1a). The $\frac{{{\mathrm{d(burned\;area)}}}}{{{\mathrm{d(VPD)}}}}$ is the slope between annual burned area and VPD for the years 2001–2020, and ranges from ~350 to 700 km^2^ hPa^−1^ (Fig. 1c), depending on ecosystem PWS. We illustrate the simplified calculation of PWS for two pixels from high- and low-PWS ecosystems in Fig. 1b. The link between PWS and $\frac{{{\mathrm{d(burned\;area)}}}}{{{\mathrm{d(VPD)}}}}$ is robust in both shrublands (*R*^2^ = 0.64, *P* = 0.005) and forests (*R*^2^ = 0.69, *P* = 0.003), but uncertain in grasslands (Supplementary Fig. 3). In grasslands, the slope between burned area and VPD is consistently low, resulting in a lower sensitivity to PWS (*R*^2^ = 0.17, *P* = 0.24).

On the basis of the nonlinear relationship between PWS and $\frac{{{\mathrm{d(burned\;area)}}}}{{{\mathrm{d(VPD)}}}}$ in Fig. 1a, we categorize PWS into three wildfire hazard categories: (1) low hazard for PWS < 1, where $\frac{{{\mathrm{d(burned\;area)}}}}{{{\mathrm{d(VPD)}}}}$ is roughly constant, (2) medium hazard for PWS $\in$ (1, 1.5), where $\frac{{{\mathrm{d(burned\;area)}}}}{{{\mathrm{d(VPD)}}}}$ increases moderately with PWS, and (3) high hazard for PWS > 1.5, where $\frac{{{\mathrm{d(burned\;area)}}}}{{{\mathrm{d(VPD)}}}}$ increases sharply with PWS. Hereafter, we refer to this definition of ‘hazard’ when interpreting our results, but use the word ‘risk’ when the hazard coincides with human exposure and vulnerability. Note that the hazard zones as defined here only refer to the increase in burned area and are not related to the losses or benefits from the area burned.

To verify that the effect of PWS on $\frac{{{\mathrm{d(burned\;area)}}}}{{{\mathrm{d(VPD)}}}}$ is causal, we test the correlation between PWS and several other potential biogeographical confounders that may correlate with PWS, but also affect $\frac{{{\mathrm{d(burned\;area)}}}}{{{\mathrm{d(VPD)}}}}$. However, we do not find any strong correlation between PWS and mean or variance of VPD, mean normalized difference vegetation index (NDVI), dry-season NDVI, or dry-season length (see Supplementary Discussion 1 and Supplementary Fig. 4). Because each bin in Fig. 1a represents a collection of locations that are disparate in space (Supplementary Fig. 2), the likelihood that other untested confounders exist is reduced.

Although we define PWS as the sensitivity of LFMC to climate-derived moisture balance, it is worth noting that this moisture balance will be influenced by VPD. Thus, VPD influences both the *x* axis and *y* axis of Fig. 1a, introducing the potential for circularity and artificially strong correlation. To evaluate this possibility, we compute a modified PWS that uses VPD as the predictor of LFMC rather than the climate-derived moisture balance. The correlation between this modified PWS and the original PWS is low (*R*^2^ = 0.13; see Supplementary Discussion 2 and Fig. 5), suggesting that VPD only weakly affects PWS relative to other factors. Thus, this potential circularity has little effect on the strong correlation in Fig. 1a, which instead is probably driven by LFMC’s effects on fire flammability and spread.

Antecedent precipitation (during the months of December–May, before the peak fire season) can spur vegetation growth, increasing fuel loads, and ultimately burned area, especially in arid ecosystems^12^. If precipitation-led growth in fuel availability is correlated with PWS, we may overestimate the role of PWS. However, we find no positive correlation between PWS and the sensitivity of fuel availability (represented by NDVI) to antecedent precipitation for any land cover (Supplementary Fig. 6). Since the effect of antecedent precipitation on fire-season fuel availability can vary with mean precipitation, we also re-compute the previous calculation across the gradient of mean precipitation, but again find no significant correlations (Supplementary Fig. 7). Overall, these sensitivity analyses suggest that the relationship in Fig. 1a is robust and probably driven by changes in LFMC dynamics across ecosystems with different PWS.

### Drivers of PWS

**Fig. 2:**
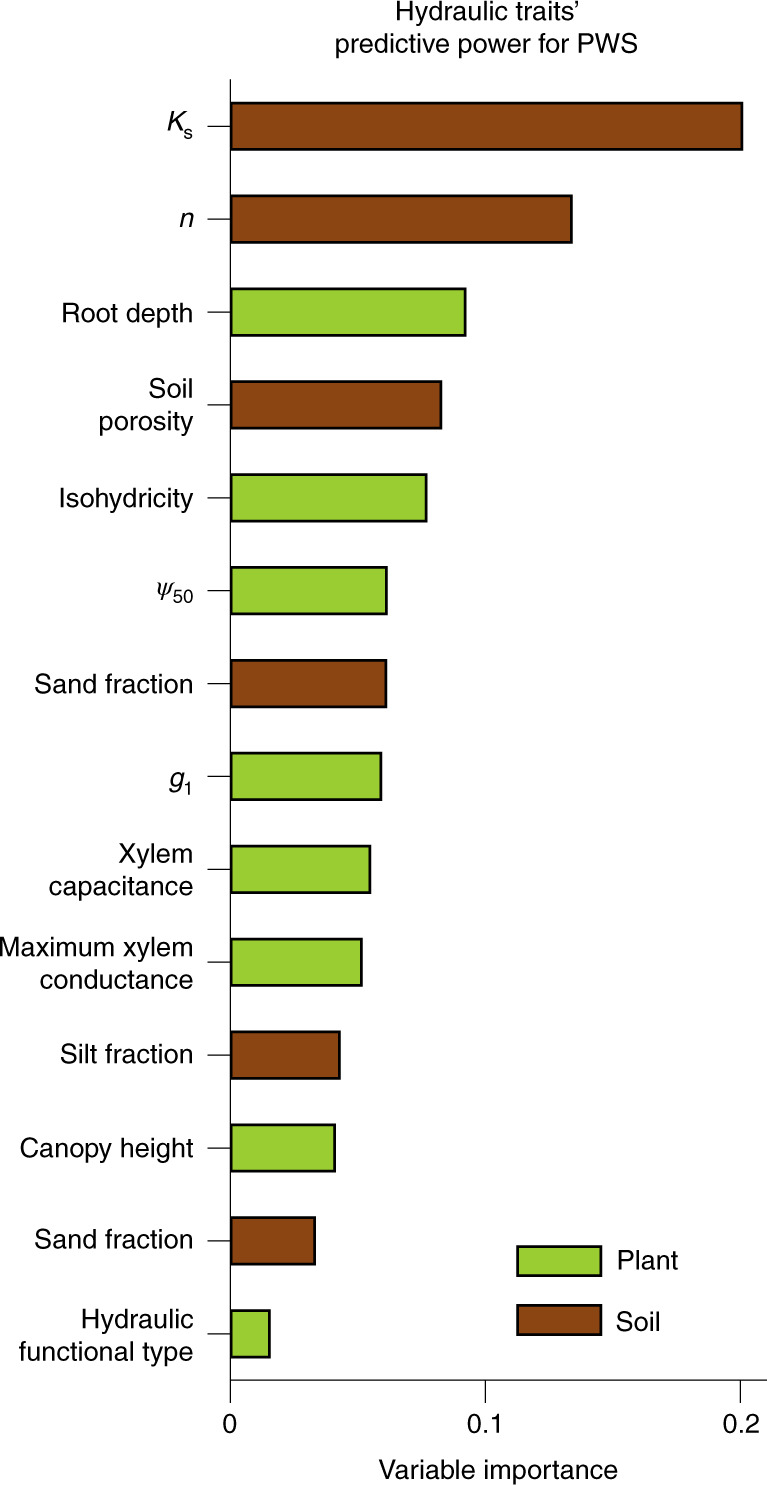
Variable importance of plant and soil hydraulic traits to predict PWS. Variable importance is estimated from average reduction in node impurity in random forests (see Methods). *K*_s_ denotes saturated soil hydraulic conductivity, *n* denotes the shape parameter of soil water retention curves^74^, *ψ*_50_ denotes xylem water potential at 50% loss in xylem conductivity, and *g*_1_ denotes stomatal conductance slope parameter from ref. ^78^, which is inversely proportional to the square root of water use efficiency. For description and data source of traits, see Supplementary Table 1.

The strong link between PWS and $\frac{{{\mathrm{d(burned\;area)}}}}{{{\mathrm{d(VPD)}}}}$ prompts the question, what drives spatial variability in PWS? Using a random forest model (see Methods), we find that 58% of the variance in PWS is explained by 14 plant and soil hydraulic traits (Supplementary Fig. 8). The most important drivers of PWS are average saturated soil hydraulic conductivity (*K*_s_, 20% importance), the shape of soil water retention curves (*n*, 13% importance) and root depth (9% importance) (Fig. 2). In total, soil and plant traits contribute to 55% and 45% of the explained importance, respectively.

### PWS is higher where VPD is increasing fastest

**Fig. 3:**
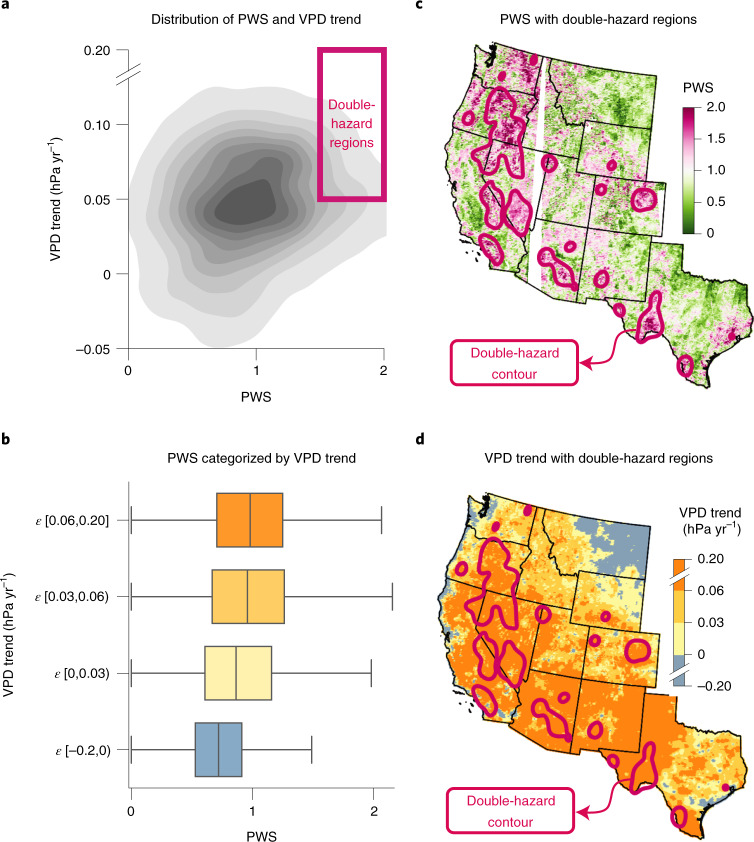
Large VPD trends and high PWS co-occur in the western US. **a**, Joint density of VPD trend from 1980–2020 and PWS. Darker colours indicate higher density. Box of double-hazard regions represents areas where wildfire hazard is high due to the co-occurrence of high PWS and VPD rise. **b**, Boxplot showing PWS distribution for four VPD trend bins. Box length indicates the interquartile range, the bisector indicates the median and whiskers extend to 1.5 times the interquartile range. The number of pixels in the four ascending VPD trend bins are 24,935, 52,591, 96,791 and 103,729, respectively. ε denotes the range in which VPD trends belong. **c**,**d** PWS map (**c**) and VPD trend map (**d**) with double-hazard region contour overlaid in pink. White patches in **c** indicate that PWS is unavailable due to insufficient data (see Methods). Black lines indicate state boundaries.

Given the role of PWS in regulating the sensitivity of burned area to VPD, we investigate how PWS has affected human exposure to wildfires. From 1980 to 2020, VPD increased across 91% of the western US, with a mean increase of 0.05 hPa yr^−1^ (Fig. 3a,d). We observe that PWS is higher in regions where VPD increased more quickly (Fig. 3a,b). The asymmetry in the joint distribution of VPD trends and PWS results in ~28% of the western US having both PWS and VPD trends that are greater than their respective median values (Fig. 3a). Results are similar when VPD trends are analysed relative to their long-term mean rather than as absolute values (Supplementary Fig. 9). Since high PWS and high VPD growth both enhance fire hazard, their co-occurrence is likely to amplify increases in burned area. This effect of ‘double-hazard’, where PWS is high (≥1.5) and VPD increased faster than average, is concentrated in regions such as the Sierra Nevada in California, eastern Oregon, the Great Basin in Nevada and the Mogollon Rim in Arizona (pink contour in Fig. 3c,d).

### The WUI has expanded disproportionately in high PWS regions

**Fig. 4:**
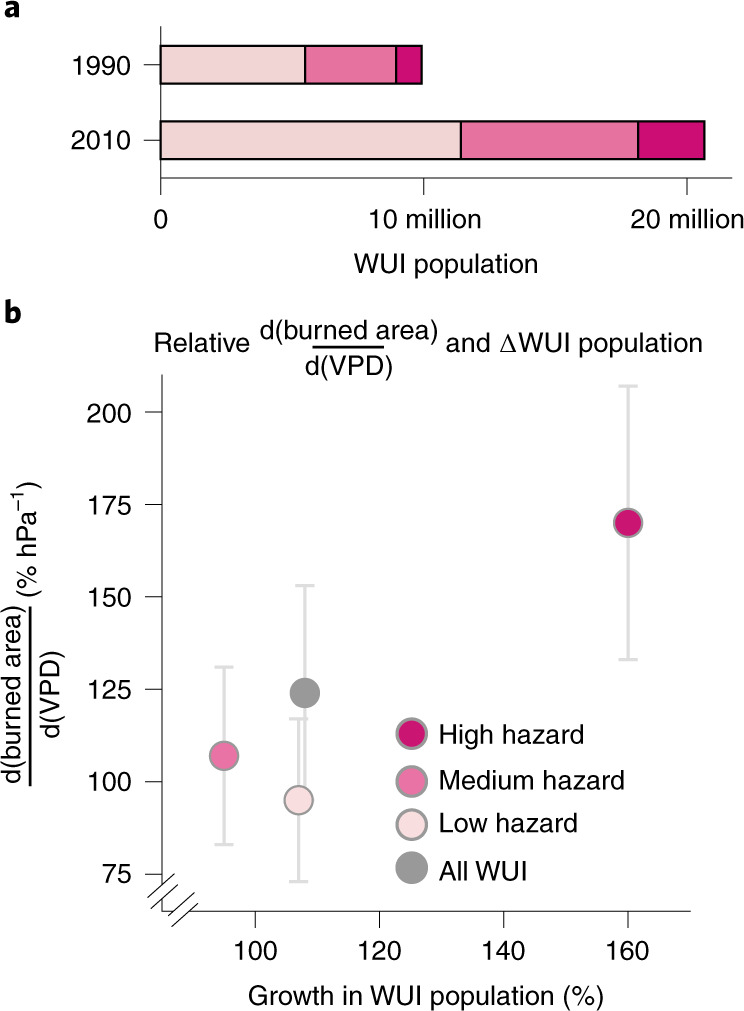
The WUI population in high wildfire hazard regions has risen at the fastest pace and experienced the most increase in percentage burned area per unit rise in VPD. **a**, WUI populations in 1990 and 2010 in each hazard zone (hazard zones shown in Fig. 1a). **b**, Percent change in burned area (relative to 2001) per unit rise in VPD versus percent change in WUI population from 1990–2010 for each hazard zone.

Beyond burned area, wildfire risk is also shaped by patterns of human exposure and vulnerability, both of which are strongly influenced by the distribution of the WUI. The WUI areas are proximal to large fuel loads, and the large number of humans and settlements in the WUI elevates both exposure and vulnerability. We thus investigate spatial patterns in WUI population growth as a function of PWS. Between 1990 and 2010, the population living in the WUI roughly doubled, growing from 10 million to 20.8 million (Fig. 4a), an increase of 108% (Fig. 4b). However, the WUI population rose most rapidly in regions with high PWS-driven wildfire hazard (160% rise). These are the same regions where burned area rose most rapidly relative to 2001 per unit rise in VPD. By contrast, in low- and medium-hazard regions, the WUI population grew by 107% and 95%, respectively.

## Discussion

The relationship in Fig. 1a suggests that vegetation regulates the effect of atmospheric aridity on burned area. This supports our hypothesis that in regions where PWS is high (for example, vegetation that keep their stomata relatively open, or have only shallow roots in soil where water infiltrates quickly), burned area increases more rapidly per unit rise in VPD compared with low-PWS regions. The larger increase in burned area in high PWS regions could be due to the greater decline in LFMC during climate-driven water limitation. Such accelerated decline in LFMC can cause a greater increase in fuel flammability or fire spread^29^, resulting in larger increase in burned area. The nonlinearity in the PWS-$\frac{{{\mathrm{d(burned\;area)}}}}{{{\mathrm{d(VPD)}}}}$ relationship could be due to a threshold-like relationship between LFMC and fuel flammability^31,32^. Despite field-scale experiments showing how plant traits and LFMC affect wildfire ignition and spread^27,33–35^, large-scale studies, especially those of burned area, have tended to ignore the effects of plant physiology, with some exceptions^18,36,37^. Although the relationship between interannual burned area and climate aridity is strong at ecoregion and landscape scales^2,38,39^, the role of vegetation in amplifying or dampening the effect of aridity on wildfires locally is still poorly understood. This is in part because LFMC is usually modelled primarily on the basis of meteorological conditions^40,41^. Given our empirical evidence that PWS – and thus, soil and plant hydraulic traits (Fig. 2) – regulate burned area variability (Fig. 1), a greater focus on the ecophysiological controls of burned area is needed to fully understand burned area drivers, both in the western US and elsewhere. Future studies that explicitly account for spatial variations in PWS or other ecophysiological controls may be better equipped for analysing and forecasting burned area.

Global fire models parameterize vegetation controls on burned area using plant functional types^42,43^. However, the plant and soil hydraulic traits that control PWS (Fig. 2) are known to vary significantly within plant functional types^44,45^. This suggests that basing vegetation–fire relationships on functional types may lead to large errors^46^. The PWS metric (1) directly quantifies vegetation-climate sensitivity, (2) is related to burned area and (3) is scalable globally. The PWS metric thus offers a potential pathway to improve global vegetation–fire modelling.

Although PWS is not a widely recognized plant trait, we show that it is an indicator of whole-plant–water relations. It combines several plant and soil hydraulic traits that affect LFMC (Fig. 2). Notably, soil hydraulic traits explain a slightly larger fraction (55% vs 45%) of the variation in PWS than do plant hydraulic traits. This is to some extent expected; datasets of the explanatory variables have much coarser resolution than our PWS dataset, and because vegetation traits are more spatially heterogeneous than soil traits, this mismatch in scales adds noise and reduces the explanatory power of the plant hydraulic traits more than that of the soil hydraulic traits. Furthermore, because PWS is determined from LFMC, a property of the vegetation, even a co-variation with soil hydraulic traits merely reflects how plant water uptake is affected by soil hydraulics. Thus, we argue that our interpretation of PWS as a metric of the water sensitivity of plants is appropriate despite the influence of soil hydraulic traits (Supplementary Discussion 3).

Vegetation drought acclimation may shift the PWS spatial pattern identified here, and thus its interaction with rising aridity and WUI population. Although drought-acclimated vegetation is likely to have lower PWS^47^, the pace of drought acclimation has probably been too slow to impact the spatial patterns of the double-hazard regions (Fig. 3) in the short term. For example, Trugman et al.^48^ showed that *ψ*_50_ of trees in the western US has reduced by ~0.001 MPa yr^−1^ between 2000 to 2019, but mean *ψ*_50_ can vary from 1 to 5 MPa geographically. Thus, the spatial variation of *ψ*_50_ is at least three orders of magnitude greater than its short-term temporal variation. In the medium-to-long term, however, major shifts in plant communities due to post-wildfire recruitment^7^ could affect the overall spatial patterns in PWS.

Our results also show that in forests and shrublands, PWS is tightly linked to wildfire hazard. The shrublands in the arid ecosystems of the southwestern US are popularly conceptualized as fuel-limited, where wildfire hazard is expected to depend on fuel availability^21,37^. However, the moderately high PWS of shrublands, combined with the lack of any significant correlation between PWS and precipitation-driven fuel growth, suggests that arid shrublands in the southwest US may also be flammability-limited in some situations (Fig. 3b and Supplementary Fig. 7). Our results thus provide a mechanistic explanation for empirical observations that previous droughts have led to a larger burned area even outside of forests in the southwestern US^49^. In grasslands, which are largely located in the Great Plains, the low slope between annual burned area and VPD suggests that other factors, such as availability of fuels, ignitions, strong winds and phenological stage, may have more dominant influences on burned area^50,51^. Since our data show that VPD played an insignificant role in governing burned area variability for grasslands (Supplementary Fig. 3), and since the temporal resolution of the LFMC dataset of 15 d may be insufficient to capture rapid responses to water limitation in grasslands, we are unable to quantify the regulating role of PWS on the response of burned area to VPD in grassland ecosystems. More generally, the overall relationship between PWS and $\frac{{{\mathrm{d(burned\;area)}}}}{{{\mathrm{d(VPD)}}}}$ has 29% unexplained variance (Fig. 1a). Studies quantifying the effect of dead fuel moisture response, vegetation demographic shifts and fire behaviour on $\frac{{{\mathrm{d(burned\;area)}}}}{{{\mathrm{d(VPD)}}}}$ may help to resolve this ambiguity.

The geographic co-occurrence of high PWS and high VPD suggests that the distribution of vegetation in the western US has amplified the effect of climate change on wildfire hazard (Fig. 3). Even though VPD has risen in most of the western US over the past few decades (Fig. 3d), PWS is higher in regions with larger VPD trends (Fig. 3b). Many of the resulting double-hazard regions with high PWS and high VPD trends are in the southwestern US. However, the PWS variations can be highly localized (Fig. 3c). For instance, while the southern Sierra Nevada has very high PWS and is among the double-hazard regions, the northern Sierra Nevada has very low PWS. Since future projections indicate continued VPD increases within the western US^16^, the spatial distribution of those VPD trends may interact with the spatial pattern of PWS to change the spatial distribution of overall fire hazard.

The expanding WUI has been viewed as a contributor to rising wildfire risk primarily due to increased human-caused ignitions^9–11,52,53^. However, as Fig. 4 demonstrates, the rise in wildfire risk to humans is also due to increasing population within high-hazard regions of the WUI. For instance, the increase in population residing in the high-hazard regions of the WUI between 1990 and 2010 (1.5 million people) is roughly equivalent to the combined current populations of San Francisco and Seattle, and represents an expansion of the share of total WUI population living in high-hazard regions from 9.7% in 1990 to 12.2% in 2010. Previous studies have recorded the growth of the WUI^8,54^, but we provide evidence that the WUI population has grown most in the most fire-vulnerable ecosystems in the western US. The disproportionate expansion of the WUI into the high PWS regions suggests that the increase in wildfire risk in the WUI is at least partly due to the higher vulnerability of vegetation to fires in particular areas of the WUI. This is corroborated by the evidence that the high-hazard zone witnessed the highest increase in relative WUI population and the highest relative $\frac{{{\mathrm{d(burned\;area)}}}}{{{\mathrm{d(VPD)}}}}$ (Fig. 4b). Further research is needed to understand the socioeconomic demographics of the populations occupying these high-hazard areas. However, at a minimum, disproportionate growth in WUI areas with high PWS and rapidly increasing VPD suggests that previous estimates of changing wildfire risk in the western US may have been conservative. While noting that PWS is not the only factor influencing wildfire risk, the PWS dataset could inform local and state-wide priorities related to wildland development, land use planning, vegetation management and home hardening solutions to curb wildfire risk.

Although wildfire risk in the western US has increased due to rising aridity and population in the WUI, the increase in risk has been significantly regulated by PWS to climate. This suggests that wildfire danger models, such as the National Fire Danger Rating System^40^, which rely on LFMC estimates derived from meteorology alone, misrepresent wildfire danger because they do not account for spatial variations in PWS. Although our analysis focuses on the western US, the effect of PWS on burned area identified here is probably also present elsewhere across the globe. Overall, the concurrence of high PWS, rising aridity and increasing WUI population has increased wildfire risk to people in many parts of the western US. In particular, the most sensitive ecosystems (PWS > 1.5) exhibit the most rapid increase in WUI population, along with widespread occurrence of above-average increase in VPD.

Accurate understanding and quantification of the processes shaping wildfire hazard, exposure and vulnerability are critical, given the growing losses from wildfire, including loss of habitat of vulnerable species^55^, loss of human life and structures^9,10^, direct and indirect economic costs^56,57^, and widespread impacts on public health beyond the area that burns^58^. Representations of wildfire risk that do not account for interactions between ecological, atmospheric and human drivers are thus susceptible to mischaracterizing wildfire risk. As we show here, examining the interplay between climate change, human population dynamics and the role of vegetation in regulating wildfire hazard can elucidate hidden interactions that lead to greater wildfire risk overall.

## Methods

### Calculating PWS

We defined PWS as the sum of slopes of a constrained linear regression between LFMC anomaly and lagged climate-derived moisture balance anomalies, with lags varying from 0 to 150 d in 15 d intervals, and a constraint that each slope be non-negative (equations 1 and 2). Because the slope between LFMC and climate-derived moisture balance is considered, PWS is an indicator of the degree to which the set of soil and plant traits in each location buffers the impact of recent and current climatic variations on LFMC. The unweighted sum of slopes was used to equally account for both concurrent and antecedent conditions. Unlike previous studies that use an unconstrained regression^59^, we constrained the regression coefficients to be non-negative. We thus did not allow an increase in climate-derived moisture balance anomaly to negatively affect an associated LFMC anomaly, which would be unphysical. Lags varied in 15 d intervals to match the temporal resolution of the LFMC data. Anomalies were computed by subtracting the pixel-specific seasonal cycle from the raw quantities to eliminate spatially varying seasonalities.

We used dead fuel moisture content (DFMC) to represent climate-derived moisture balance. The DFMC is an indicator of wetness and combines precipitation, temperature and humidity over lagged timescales into one index. It is sensitive to not just demand-side components of the water balance, such as atmospheric moisture demand, but also supply-side components, such as precipitation^60^. Here we used the 100 h DFMC, which is a meteorological estimate of wetness of twigs and branches 1–3 inches in diameter. It is calculated from the 24 h antecedent precipitation and the equilibrium moisture content corresponding to the drying–wetting potential of the atmosphere during the same period after adjusting for the duration of daylight^61^.

We calculated PWS as follows.

1 $${{{\mathrm{PWS}}}}_s = \mathop {\sum }\limits_{{{i}}} {\beta}_{{{s}},{{{i}}}}$$

2 $${{{\mathrm{LFMC}}}}^\prime _{{{{s}}},{{{t}}}} = \mathop {\sum }\limits_{{{{i}}} = 0,15,30 \ldots }^{{{{i}}} = 150} {\beta}_{{{{s}}},{{i}}} \times {{{\mathrm{DFMC}}}}_{{{{t}}} - {{{i}}},{{{s}}}}^\prime + \gamma _s;\;{\beta}_{{{{s}}},{{{i}}}} \ge 0$$

where,

3 $${{{\mathrm{LFMC}}}}^\prime _{{{{s}}},{{{t}}}} = {{{\mathrm{Live}}}}\;{{{\mathrm{fuel}}}}\;{{{\mathrm{moisture}}}}\;{{{\mathrm{content}}}}\;{{{\mathrm{anomaly}}}} = {{{\mathrm{LFMC}}}}_{{{{s}}},{{{t}}}} - \overline {{{{\mathrm{LFMC}}}}_{{{{s}}},{{{\mathrm{doy}}}}({{{t}}})}}$$

DFMC′ is the dead fuel moisture content anomaly (computation same as LFMC′), *t* is the date between June to November for the years 2016–2020, doy is day of year, *s* is the pixel index, *γ* is the intercept, *β* is the slope and *i* is the lag between LFMC′ and DFMC′ in number of days. The *i* increases in steps of 15 d intervals (half-monthly). The $\overline {{{{\mathrm{LFMC}}}}_{{{{s}}},{{{\mathrm{doy}}}}({{{t}}})}}$ represents the mean LFMC at pixel *s*, on day of year *t* (across 2016–2020).

According to equations (1) and (2), if LFMC purely reflects climate-derived moisture balance, the PWS is high, whereas if LFMC is buffered against climatic water limitation (for example, through regulatory mechanisms such as stomatal control, root water uptake and so on^62^), PWS is low. We used data from 2016 to 2020 to estimate PWS. We limited our observations to June–November to focus on the period during which plant water dry-down occurs due to climatic water limitation. By limiting our observations to this period, the sensitivity of PWS to other processes, such as plant growth in the spring, is minimized. We commenced our analysis in 2016, the first year of LFMC data availability. The PWS was not computed for the 9.5% of pixels where more than 50% of the LFMC temporal record was missing due to cloud or snow cover, or where synthetic aperture radar data was absent. A maximum plant water ‘memory’ of 150 d was chosen due to the apparent hydrological memory timescales of ecosystems of the western US^63^. The linear regression between LFMC′ and DFMC′ has a mean *R*^2^ of 0.3, with a wide distribution of fits (*R*^2^ ranging from 0 to 0.85; Supplementary Fig. 10). A relatively low mean *R*^2^ of 0.3 is consistent with expectations, as plant and soil hydraulic traits that contribute to plant water control are known to cause significant differences between LFMC and meteorology^62,64,65^. Since the regression coefficients were constrained to be non-negative, ~50% of the coefficients are zero (Supplementary Fig. 11).

We used LFMC data from an artificial intelligence model trained and validated in ecosystems of the western US using microwave and optical remote sensing^30^. The LFMC dataset was trained on field-measured moisture content of live leaves, needles and thin branches from the National Fuel Moisture Database^66^. The dataset has an overall *R*^2^ = 0.63, RMSE = 25% and bias = −1.9%, with consistent performance across all land cover types. We used DFMC data from GRIDMET^67^. We resampled the DFMC data to 15 d averages to match the start and end times of the LFMC maps. We rescaled the LFMC dataset from 250 m to 4 km using bilinear interpolation to match the spatial resolution of the DFMC dataset.

### Linking PWS to the sensitivity of burned area to VPD

We separated the PWS map into 15 regions with equal-vegetated area on the basis of the histogram of PWS (Supplementary Fig. 1). Vegetated areas included regions with trees, shrubs and grasslands obtained from the National Land Cover Database version 2016^68^. We then computed the slope between burned area and VPD in each region using linear regression. Combining different locations of similar PWS enabled calculating the burned area, which otherwise was not possible at the pixel-level. Our results were consistent when PWS was separated into 10 regions instead of 15 (Supplementary Fig. 12). The linear fits between burned area and VPD have *R*^2^ ranging from 0.48 to 0.58 and *P* values ranging from 0.0001 to 0.0010.

Although many aridity metrics have been used to study interannual changes in burned area^2,37^, we chose VPD to represent atmospheric aridity because of its parsimonious definition, its direct connection to atmospheric moisture demand, and its strong link to burned area compared with that of other meteorologically based aridity indicators^49,69^. Further, by not choosing the same meteorological indicator as the one chosen to assess the response of PWS to climate-derived moisture balance (DFMC), we minimized the introduction of artificial cross-correlations.

For each of the 15 PWS bins, we computed the slope between annual burned area and mean annual VPD, each calculated across April to March. We computed the slope using linear regression with an unconstrained intercept. We used burned area data for 2001–2020 from the Moderate Resolution Imaging Spectroradiometer burned area product MCD64A1^70^, and VPD data from the Parameter-elevation Regressions on Independent Slopes Model^71^. We aggregated burned area from 500 m to 4 km resolution to match the resolution of VPD. We then computed the slopes and their associated linear standard errors for each of the 15 PWS bins, followed by computation of a final linear regression between $\frac{{{\mathrm{d(burned\;area)}}}}{{{\mathrm{d(VPD)}}}}$ and PWS (Fig. 1a).

### Spatial drivers of PWS

To investigate what factors influence PWS, we regressed the PWS map against eight plant hydraulic traits and six soil hydraulic traits using an ensemble approach. For plant traits, we used canopy height from 2005 from ref. ^72^ and several hydraulic traits, including xylem capacitance, a stomatal conductance slope parameter that is inversely proportional to marginal water use efficiency (*g*_1_), maximum xylem conductance, the *ψ*_50_, and a hydraulic functional type derived from *K*-means clustering of these plant hydraulic traits determined from ref. ^41^. We also included isohydricity (a stomatal regulation trait) from ref. ^45^, and maximum rooting depth from ref. ^73^. For soil traits, we used average saturated hydraulic conductivity (*K*_s_) the shape parameter describing the exponent of soil water retention curves (*n*), soil porosity from ref. ^74^, and fraction of soil, silt and clay from ref. ^75^ (Supplementary Table 1). We rescaled all traits to 4 km resolution using nearest neighbour interpolation since all traits (except for canopy height and rooting depth) have a coarse resolution of 25 km.

We used the rescaled traits as explanatory variables in a random forest regression to assess the importance of each trait. We ignored all time-varying factors in our analysis and used static maps of all traits to explain the variance in the static map of PWS. We used 3-fold cross-validation to evaluate strength of fit (Supplementary Fig. 8). To compute variable importance, we measured the average decrease in node impurity due to splitting at each variable and normalized the output (Fig. 2). For the random forest ensembles, we chose a minimum of six samples per terminal node, a minimum of 5 × 10^−6^ reduction in node impurity at each split, a minimum of two samples split per node, bootstrapped samples during node splits, and 50 trees within each forest. We used scikit-learn for the random forest regression^76^.

### Estimating VPD trends

We estimated VPD trends using a pixel-specific linear regression of the annual-average VPD against year, for the period 1980–2020. For further investigation, we calculated relative VPD trends by dividing the absolute VPD trends by the pixel-specific 1980–2020 VPD mean (Supplementary Fig. 9).

### Estimating WUI expansion

We used maps of population density and WUI from Martinuzzi et al.^77^ to track WUI expansion from 1990 to 2010 in regions with different wildfire hazard. Martinuzzi et al. identified WUI regions using pre-defined thresholds for density of human settlements in wildland areas, with different thresholds for intermix (≥6.18 houses per km^2^ and ≥50% cover of wildland vegetation) and interface regions (≥6.18 houses per km^2^ and <50% cover of vegetation, located <2.4 km of an area ≥5 km^2^ in size that is ≥75% vegetated). We did not differentiate between WUI intermix and WUI interface regions since both represent regions with high wildfire risk. We rescaled the WUI maps from their original census block-level resolution to 4 km to match the resolution of PWS. We classified any 4 km pixel that overlaps with a block-level WUI polygon as WUI since it contains at least some area with neighbouring urban and vegetated areas (Supplementary Fig. 13).

### Reporting Summary

Further information on research design is available in the Nature Research Reporting Summary linked to this article.

## Supplementary information

Supplementary Information Supplementary Figs. 1–13, Table 1 and Discussions 1–3.

Reporting Summary

Peer Review Information

## Data Availability

The PWS map is available at https://github.com/kkraoj/wildfire_from_lfmc. The LFMC maps are available at https://kkraoj.users.earthengine.app/view/live-fuel-moisture. Climate data from GRIDMET are available at http://www.climatologylab.org/gridmet.html. Wildland–urban interface maps are available at http://silvis.forest.wisc.edu/data/wui-change/.

## Code availability

The scripts required to reproduce our results are available at https://github.com/kkraoj/wildfire_from_lfmc.
