# Supplementary material for: Plant-water sensitivity regulates wildfire vulnerability
Source: Nat Ecol Evol. 2022 Feb 7;6(3):332–9. doi: 10.1038/s41559-021-01654-2 (PMC8913365; doi:10.1038/s41559-021-01654-2)
Supplement: Supplementary file 1 — Supplementary Figs. 1–13, Table 1 and Discussions 1–3. [file 41559_2021_1654_MOESM1_ESM.pdf]

nature portfolio
https://doi.org/10.1038/s41559-021-01654-2

Supplementary information

Plant-water sensitivity regulates wildfire vulnerability

In the format provided by the
authors and unedited

Supporting information for

Plant Water Sensitivity Regulates Wildfire Vulnerability

Krishna Rao, A. Park Williams, Noah S. Diffenbaugh, Marta Yebra, Alexandra G. Konings

This file contains:

Supplementary Figures 1 to 13

Supplementary Table 1

Supplementary Discussion 1 to 3

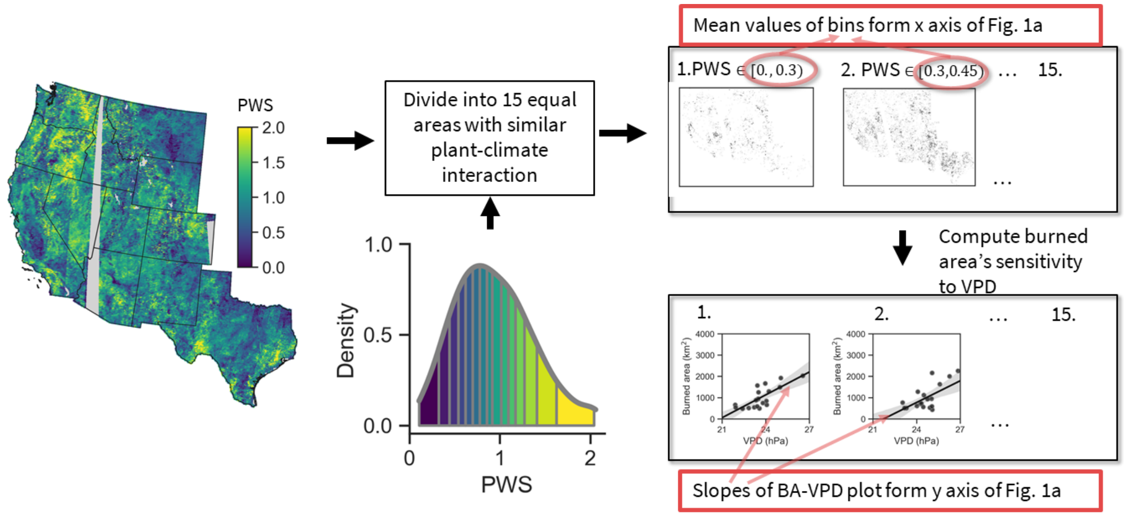

Supplementary Fig. 1 | Process chart to prepare Fig. 1A. The western US is segregated into 15 equal-vegetated area regions based on the plant-water sensitivity (PWS) histogram. For each region, the slope of burned area (BA) versus VPD, and mean PWS is noted. Finally, the two are plotted against each other to make Fig. 1A. Grey patches in the PWS map indicate areas where PWS is unavailable due to missing Sentinel-1 data, snow cover, or cloud cover (see Methods).

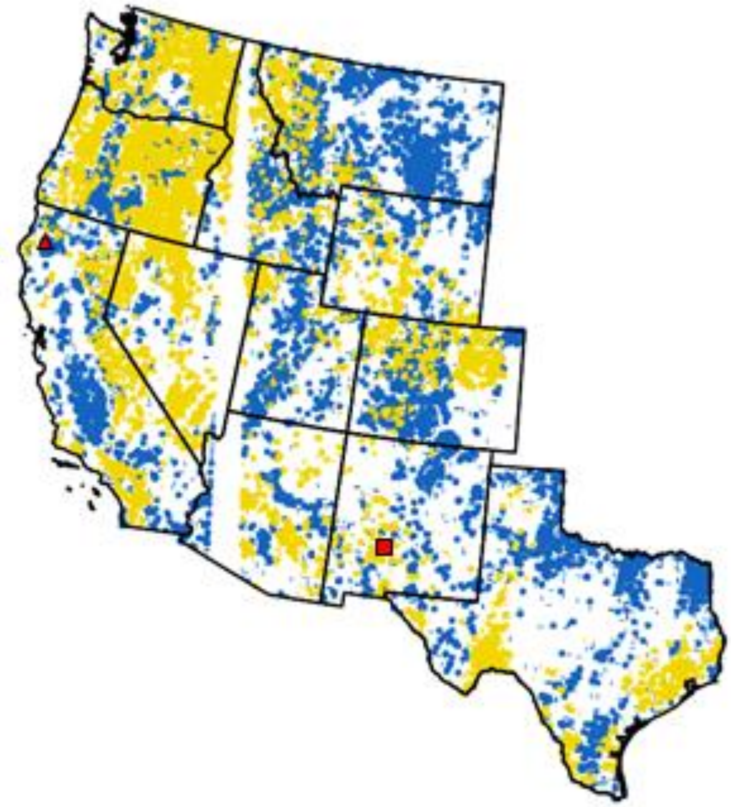

Supplementary Fig. 2 | First (blue) and last (yellow) plant-water sensitivity (PWS) bins from Fig. 1c are shown. Red triangle (square) indicates the location of example pixel within first (last) PWS bin which was chosen to represent PWS calculation in Fig. 1b.

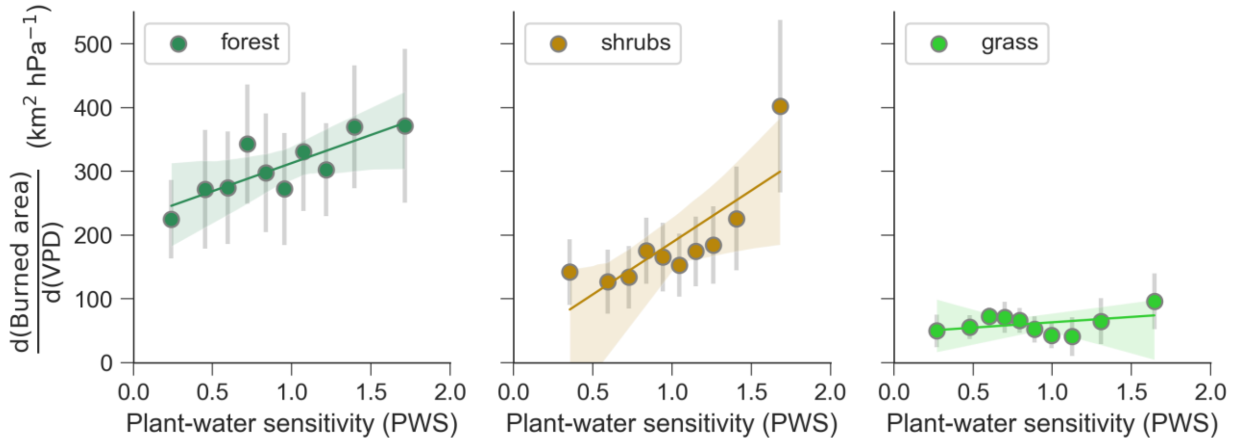

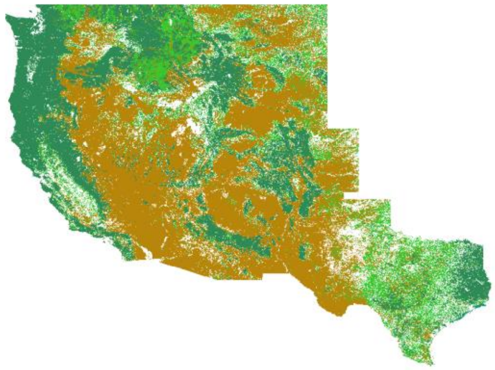

Supplementary Fig. 3 | Sensitivity of burned area to VPD is correlated with plant-water sensitivity (PWS) in shrublands and forest. For shrublands R^2=0.64, p=0.005, forests R^2=0.69, p=0.003, and for grasslands R^2=0.17, p=0.24. Points indicate data for 10 equal-vegetated area bins of PWS and are colored by land cover Vertical grey bars indicate 1 standard error in estimate of slope between burned area and VPD. Colored line indicates the best fit, with shaded band showing 95% confidence interval. Land cover map for 2016 from the National Land Cover Database (C. G. Homer et al., 2011) shown in the bottom row. The land cover map is based on inputs from Landsat data. The dataset has been validated extensively in the USA with an overall area-weighted accuracy of 82% (C. Homer & Fry, 2012).

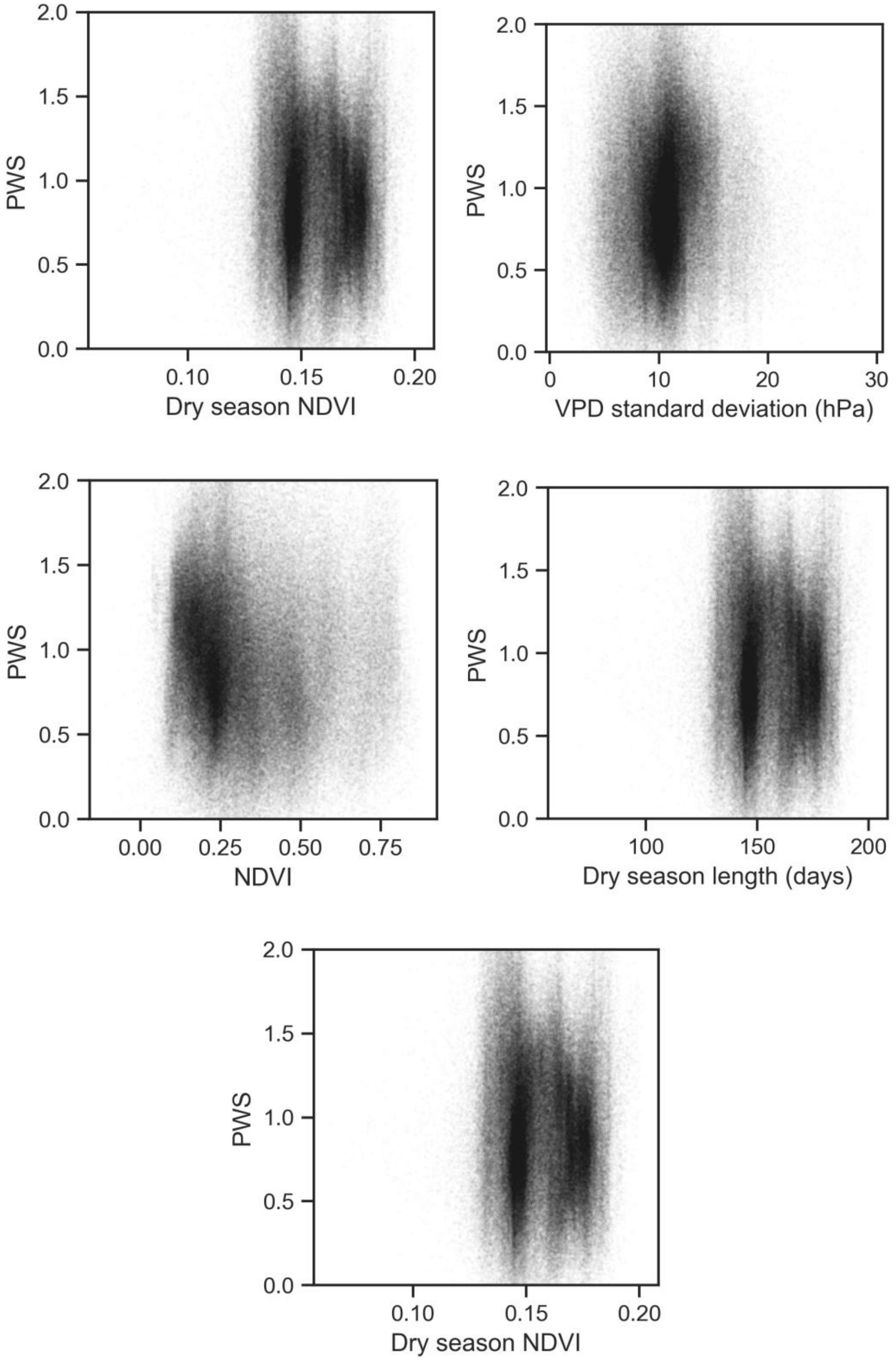

Supplementary Fig. 4 | Scatterplot of plant-water sensitivity (PWS) against mean VPD, standard deviation of VPD, normalized difference vegetation index (NDVI), dry season length (calculated as the average number of days in a year during which VPD>historic

mean VPD for each pixel using PRISM data record from 1980 – 2020), and dry season NDVI (Jun to Nov). All correlations have R2 < 0.006.

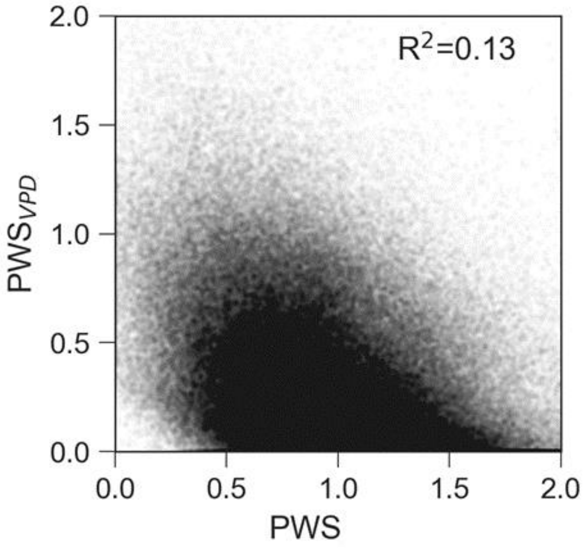

Supplementary Fig. 5 | Relationship between plant-water's sensitivity to atmospheric moisture demand (PWS_{VPD}) and plant-water's sensitivity to climate-derived moisture balance (PWS). See Sec. V.a. and Supplementary Discussion 2 for equations to calculate PWS and PWS_{VPD}, respectively.

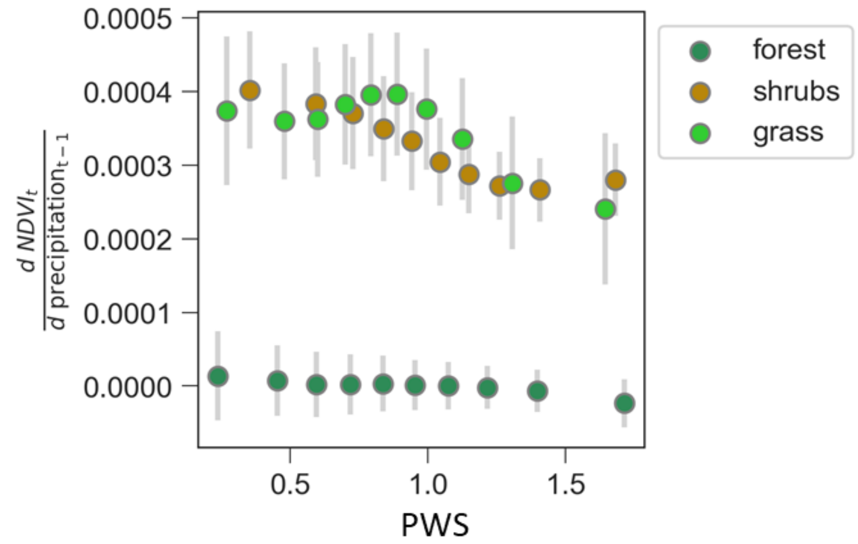

Supplementary Fig. 6 | Plot showing no positive correlation between plant-water sensitivity (PWS) and sensitivity of fire season's (Jun – Nov) fuel availability ($NDVI_t$) to antecedent (Dec – May) precipitation (year t-1) for all three land cover types. The NDVI is used as a proxy for fuel availability.

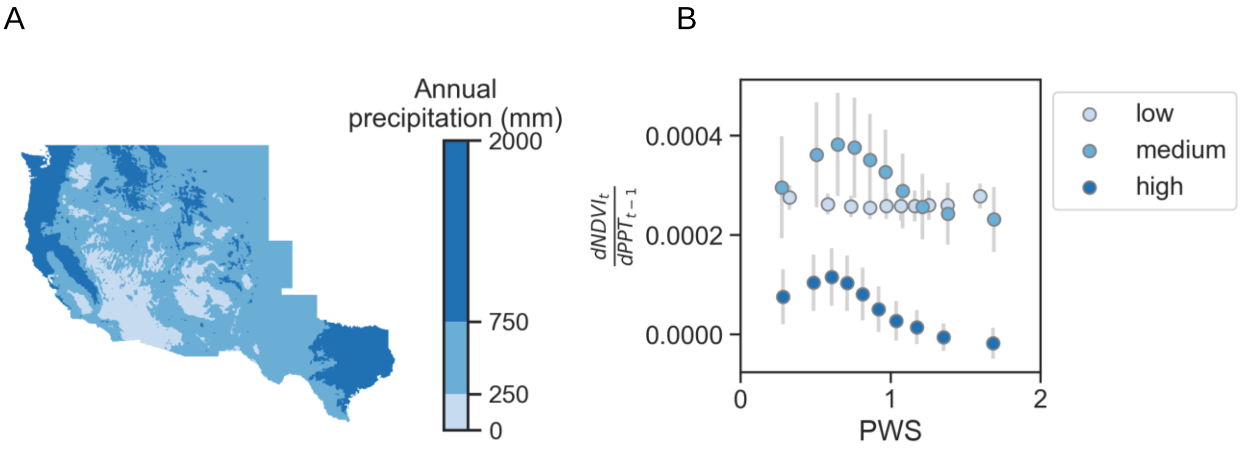

Supplementary Fig. 7 | (A) Map of annual precipitation for the period 1980 - 2020. Precipitation data obtained from PRISM (PRISM Climate Group Oregon State University, 2004). (B) Plot showing no positive correlation between plant-water senility (PWS) and sensitivity of fire season's (Jun – Nov) fuel availability ($NDVI_t$) to antecedent (Dec – May) precipitation (year t-1) for all three precipitation zones (as defined in sub-panel A).

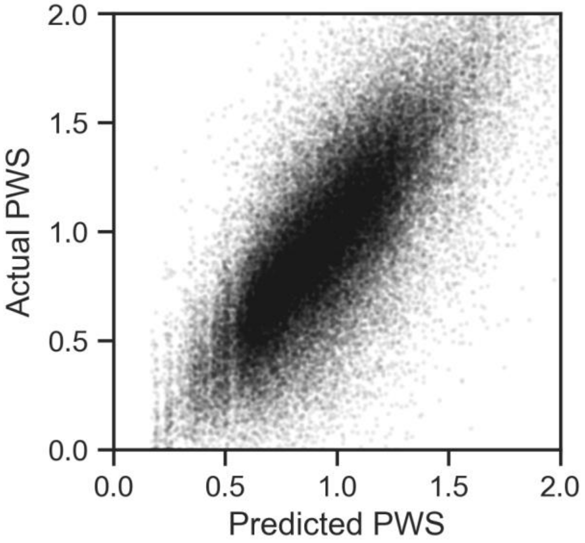

Supplementary Fig. 8 | Performance of the random forest regression on leave-out validation set. "Actual PWS" refers to the PWS used in the manuscript. "Predicted PWS" refers to the PWS from the random forest regression (see Methods). R^2 for the fit is 0.58.

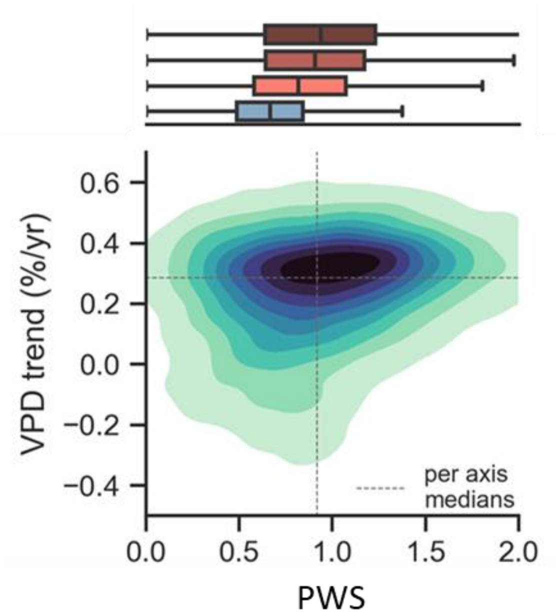

Supplementary Fig. 9 | Large VPD relative trends (in %) and high plant-water sensitivity (PWS) have co-occurred in the western US. Joint distribution of relative VPD trend and PWS shows the asymmetrical nature of the distribution with the bulk of the mass in the top right quadrant. (Above) Box plot of PWS distribution for four VPD bins defined by [-1,0), [0,0.25), [0.25,0.5), [0.5,1] shows that the median PWS is higher for regions with higher relative trends in VPD. Box length indicates the interquartile range. Box bisector indicates the median. Whiskers extend to 1.5 times interquartile range.

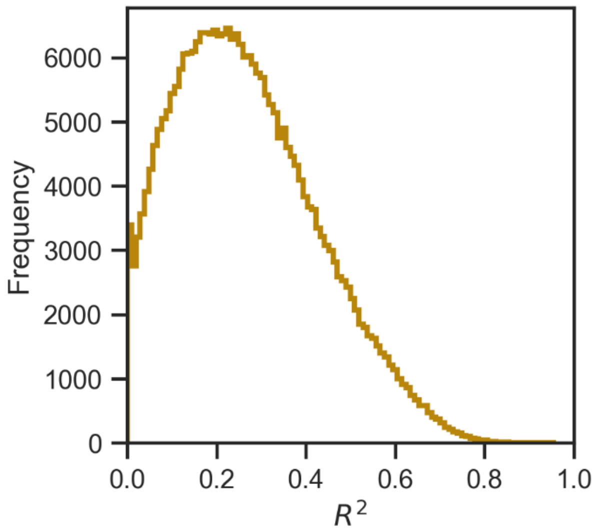

Supplementary Fig. 10 | Histogram of the strength of multiple linear regression fits used to calculate plant-water sensitivity (PWS). Y axis is number of pixels.

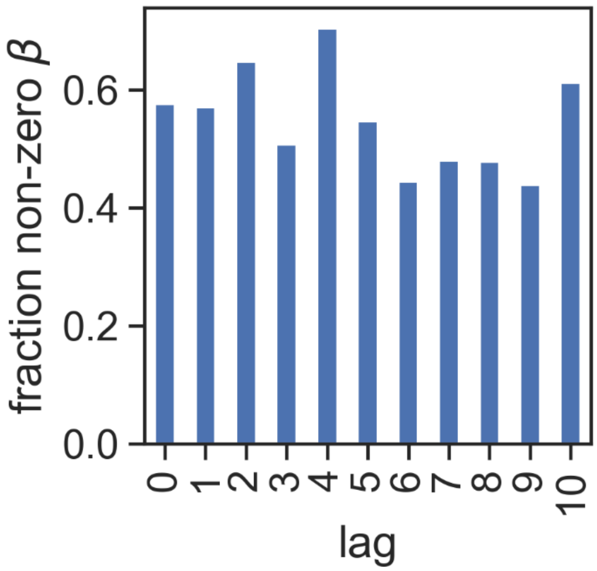

Supplementary Fig. 11 | Fraction of non-zero coefficients in linear regression used to calculate PWS (as per equation 1). The β refers to the slope for each lagged DFMC'.

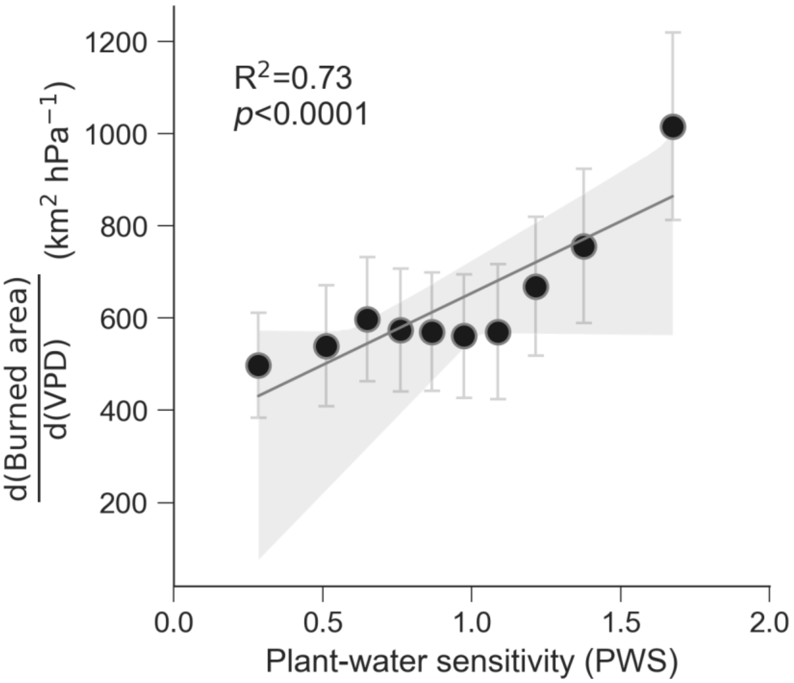

Supplementary Fig. 12 | Sensitivity of burned area to VPD is correlated with PWS. Black points indicate data for 10 equal-vegetated area bins of PWS. Vertical grey bars indicate 1 standard error in the estimate of slope between burned area and VPD. Thick grey line indicates the best fit, with a shaded band showing the 95% confidence interval.

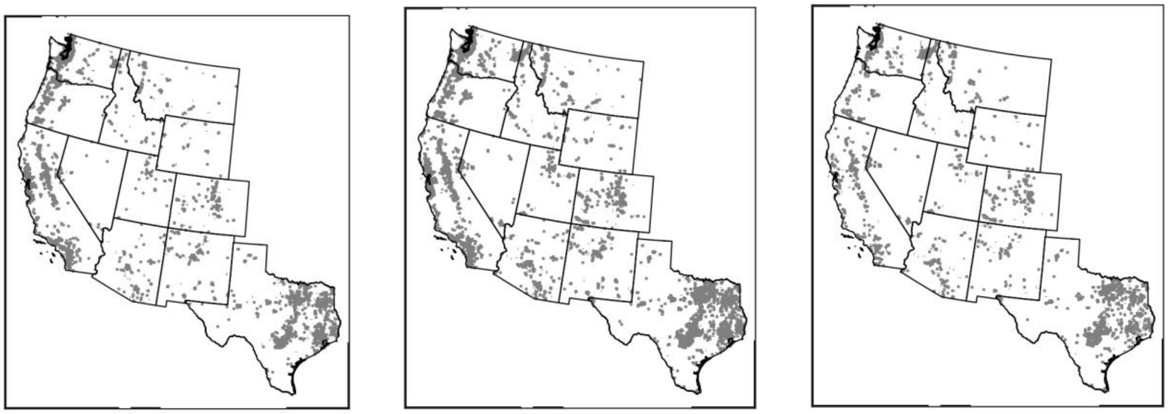

Supplementary Fig. 13 | WUI areas in 1990 (left) and 2010 (middle) and the difference between WUI in 1990 and 2010 (right) rescaled to 4 km resolution.

Supplementary Table 1 | Plant and soil hydraulic triats used in random forest regression to evaluate spatial drivers of PWS.

Category	*Variable Name*	*Description*	*Source*
Vegetation	Canopy height	Height of canopy	(Simard et al. 2011)
	Xylem capacitance	Proportionality between water flux and the corresponding change in xylem potential	(Liu et al. 2021)
	g_1	Slope parameter in (Medlyn *et al.* 2011) which is inversely proportional to square root of water use efficiency. This is a stomatal trait.	(Liu et al. 2021)
	Max. xylem conductance	Maximum xylem conductance	(Liu et al. 2021)
	Hydraulic functional type	Derived from K-means clustering of plant traits	(Liu et al. 2021)
	ψ_{50}	Xylem water potential at 50% loss of xylem conductivity	(Liu et al. 2021)
	Isohydricity	Level of stomatal and xylem regulation	(Konings & Gentine 2017)
	Rooting depth	Maximum rooting depth	(Fan et al. 2017)
Soil	K_s	Average saturated hydraulic conductivity	(Montzka et al. 2017)
	Porosity	Average saturated volumetric water content of soil (soil porosity)	(Montzka et al. 2017)
	n	Shape parameter in soil water retention curve. This is the exponent in the pedo-transfer function in the van Genuchten model.	(Montzka et al. 2017)
	Silt fraction	Fraction of silt content in soil	(Liu et al. 2014)
	Sand fraction	Fraction of sand content in soil	(Liu et al. 2014)
	Clay fraction	Fraction of clay content in soil	(Liu et al. 2014)

Supplementary Discussion 1

The strong link between $\frac{d\ burned\ area}{d\ VPD}$ and PWS could be the result of vegetation's regulating role on the sensitivity of burned area to VPD, or merely due to confounders that are correlated with PWS, but also affect $\frac{d\ burned\ area}{d\ VPD}$. To ensure the relationship in Fig. 1A is causal, PWS was compared to several biogeographic variables that might influence $\frac{d\ burned\ area}{d\ VPD}$ (Supplementary Fig. 3). However, we do not find any significant correlations between PWS and mean VPD, intra-year standard deviation of VPD (a measure of climate extremes), length of the dry season, NDVI (an indicator of fuel availability; Tucker 1979), or dry-season NDVI (Jun to Nov). All correlations have $R^2<0.006$.

Supplementary Discussion 2

To verify whether PWS indeed reflects plant-water's sensitivity to climate-derived moisture balance and not just plant-water's sensitivity to atmospheric aridity, we replicate the computation of PWS as in equations 1 and 2, but use VPD instead of DFMC. That is,

$$\mathrm{PWS}_{VPD} = \sum_{\mathrm{i}} \beta_{\mathrm{s,i}} \qquad \text{–(4)}$$

$$\mathrm{LFMC}'_{\mathrm{s,t}} = \sum_{\mathrm{i=0,15,30\ldots}}^{\mathrm{i=150}} \beta_{\mathrm{s,i}} \times \mathrm{VPD}'_{\mathrm{t-i,s}} + \gamma_s\,;\ \beta_{\mathrm{s,i}} \geq 0 \qquad \text{–(5)}$$

where VPD' = Vapor pressure deficit anomaly, and all other variables follow from equation (3).

We posit that the PWS_{VPD} will not yield the same values as PWS (as calculated from equation (1)). Comparing PWS_{VPD} with PWS, we observe that the two are not strongly related (R^2=0.13, Supplementary Fig. 8), indicating that PWS indeed quantifies plant-water's sensitivity to climate-derived moisture balance and not just to atmospheric moisture demand.

Supplementary Discussion 3

Plant and soil hydraulic traits both influence PWS. Since PWS is derived from LFMC's sensitivity to lagged climate-derived moisture balance (see Methods), all hydraulic traits that affect LFMC are expected to influence PWS too. We broadly categorize all hydraulic traits as soil traits or plant traits (Supplementary Table 1), but each trait affects plant moisture in different stages of the soil-plant continuum: from root access to soil water, to water transport through the xylem and leaves, and finally to transpiration. Our ability to identify the importance of the hydraulic traits belonging to each of the stages is limited by data quality. Thus, the relative importance of the variables used in the random forest regression is affected by at least three factors. First, accuracy of data on soil hydraulic traits is higher than plant hydraulic traits. Soil hydraulic traits are derived from intensively sampled in-situ measurements and sub-1-km scale data (Montzka et al. 2017). By contrast, most of the plant hydraulic traits we use are retrieved from data assimilation of satellite observables (Liu et al. 2021), which are sensitive to errors in the plant hydraulic model underlying the data assimilation, remote sensing retrieval algorithm errors, and uncertainties in the data assimilation methodology. The magnitude of these uncertainties is reflected in the considerable magnitude of the estimated uncertainty for these hydraulic traits (Liu et al., 2021). Second, there is a large mismatch in the spatial resolution of the hydraulic traits and PWS. All hydraulic traits except for canopy height (300 m resolution, though derived from a ten-year old dataset) and root depth (1 km resolution) are available only at 25 km resolution, whereas PWS is available at 4 km resolution. The spatial mismatch may have adversely impacted the importance of plant hydraulic traits, since they are known to be extremely heterogenous (Anderegg 2015). By contrast, the effect of this

mismatch should be smaller for soil hydraulic traits, which are generally more homogenous (Liu et al. 2014). Third, 43% of the variation in PWS is unaccounted for in our analysis. The unexplained variance might be due to other plant hydraulic traits which are not included in our analysis (Trugman et al. 2019). All three above-mentioned factors combined with the near-equal influence of soil and plant hydraulic traits on PWS (Fig. 2) suggests PWS represents whole-plant water relations and not just soil-water relations alone.

References

Anderegg, W. R. L. (2015). Spatial and temporal variation in plant hydraulic traits and their relevance for climate change impacts on vegetation. *New Phytologist*. https://doi.org/10.1111/nph.12907

Fan, Y., Miguez-Macho, G., Jobbágy, E. G., Jackson, R. B., & Otero-Casal, C. (2017). Hydrologic regulation of plant rooting depth. *Proceedings of the National Academy of Sciences of the United States of America*, *114*(40), 10572–10577. https://doi.org/10.1073/pnas.1712381114

Homer, C., & Fry, J. (2012). The National Land Cover Database. *US Geological Survey Fact Sheet*, (February), 1–4.

Homer, C. G., Dewitz, J. A., Yang, L., Jin, S., Danielson, P., Xian, G., et al. (2011). Completion of the 2006 National Land Cover Database for the conterminous United States. *Photogrammetric Engineering and Remote Sensing*, *77*, 858–566. Retrieved from https://www.cabdirect.org/cabdirect/abstract/20113310420

Konings, A. G., & Gentine, P. (2017). Global variations in ecosystem-scale isohydricity. *Global Change Biology*, *23*(2), 891–905. https://doi.org/10.1111/gcb.13389

Liu, S., Wei, Y., Post, W. M., Cook, R. B., Schaefer, K., & Thorton, M. M. (2014). NACP MsTMIP: Unified North American Soil Map. ORNL Distributed Active Archive Center. https://doi.org/10.3334/ornldaac/1242

Liu, Y., Holtzman, N. M., & Konings, A. G. (2021). Global ecosystem-scale plant hydraulic traits retrieved using model-data fusion. *Hydrology and Earth System Sciences*, *25*(5), 2399–2417. https://doi.org/10.5194/hess-25-2399-2021

Medlyn, B. E., Duursma, R. A., Eamus, D., Ellsworth, D. S., Prentice, I. C., Barton, C. V. M., et al. (2011). Reconciling the optimal and empirical approaches to modelling stomatal conductance. *Global Change Biology*, *17*(6), 2134–2144. https://doi.org/10.1111/j.1365-2486.2010.02375.x

Montzka, C., Herbst, M., Weihermüller, L., Verhoef, A., & Vereecken, H. (2017). A global data set of soil hydraulic properties and sub-grid variability of soil water retention and hydraulic conductivity curves. *Earth System Science Data*, *9*(2), 529–543. https://doi.org/10.5194/essd-9-529-2017

PRISM Climate Group Oregon State University. (2004). PRISM Climate Data. https://doi.org/20 May 2016

Simard, M., Pinto, N., Fisher, J. B., & Baccini, A. (2011). Mapping forest canopy height globally with spaceborne lidar. *Journal of Geophysical Research: Biogeosciences*, *116*(4), 1–12. https://doi.org/10.1029/2011JG001708

Trugman, A. T., Anderegg, L. D. L., Sperry, J. S., Wang, Y., Venturas, M., & Anderegg, W. R. L. (2019). Leveraging plant hydraulics to yield predictive and dynamic plant leaf allocation in vegetation models with climate change. *Global Change Biology*, *25*(12), 4008–4021. https://doi.org/10.1111/gcb.14814

Tucker, C. J. (1979). Red and photographic infrared linear combinations for monitoring vegetation. *Remote Sensing of Environment*, *8*(2), 127–150. https://doi.org/10.1016/0034-4257(79)90013-0
