# Supplementary material for: Plant-water sensitivity regulates wildfire vulnerability
Source: Nat Ecol Evol. 2022 Feb 7;6(3):332–9. doi: 10.1038/s41559-021-01654-2 (PMC8913365; doi:10.1038/s41559-021-01654-2)
Supplement: Supplementary file 2 — Reporting Summary [file 41559_2021_1654_MOESM2_ESM.pdf]

nature portfolio

Corresponding author(s): Krishna Rao

Last updated by author(s): Dec 11, 2021

Reporting Summary

Nature Portfolio wishes to improve the reproducibility of the work that we publish. This form provides structure for consistency and transparency in reporting. For further information on Nature Portfolio policies, see our Editorial Policies and the Editorial Policy Checklist.

Statistics

For all statistical analyses, confirm that the following items are present in the figure legend, table legend, main text, or Methods section.

n/a	Confirmed	
☒	☐	The exact sample size (*n*) for each experimental group/condition, given as a discrete number and unit of measurement
☒	☐	A statement on whether measurements were taken from distinct samples or whether the same sample was measured repeatedly
☒	☐	The statistical test(s) used AND whether they are one- or two-sided<br>*Only common tests should be described solely by name; describe more complex techniques in the Methods section.*
☐	☒	A description of all covariates tested
☐	☒	A description of any assumptions or corrections, such as tests of normality and adjustment for multiple comparisons
☐	☒	A full description of the statistical parameters including central tendency (e.g. means) or other basic estimates (e.g. regression coefficient) AND variation (e.g. standard deviation) or associated estimates of uncertainty (e.g. confidence intervals)
☐	☒	For null hypothesis testing, the test statistic (e.g. *F*, *t*, *r*) with confidence intervals, effect sizes, degrees of freedom and *P* value noted<br>*Give P values as exact values whenever suitable.*
☒	☐	For Bayesian analysis, information on the choice of priors and Markov chain Monte Carlo settings
☒	☐	For hierarchical and complex designs, identification of the appropriate level for tests and full reporting of outcomes
☒	☐	Estimates of effect sizes (e.g. Cohen's *d*, Pearson's *r*), indicating how they were calculated

Our web collection on statistics for biologists contains articles on many of the points above.

Software and code

Policy information about availability of computer code

Data collection	https://github.com/kkraoj/wildfire_from_lfmc/tree/master/misc
Data analysis	https://github.com/kkraoj/wildfire_from_lfmc/tree/master/analysis

For manuscripts utilizing custom algorithms or software that are central to the research but not yet described in published literature, software must be made available to editors and reviewers. We strongly encourage code deposition in a community repository (e.g. GitHub). See the Nature Portfolio guidelines for submitting code & software for further information.

Data

Policy information about availability of data

All manuscripts must include a data availability statement. This statement should provide the following information, where applicable:

- Accession codes, unique identifiers, or web links for publicly available datasets
- A description of any restrictions on data availability
- For clinical datasets or third party data, please ensure that the statement adheres to our policy

The derived plant-water sensitivity map along with the scripts required to reproduce the results presented in this manuscript can be found at https://github.com/kkraoj/wildfire_from_lfmc. Live fuel moisture content maps are available from https://kkraoj.users.earthengine.app/view/live-fuel-moisture. Climate data from GRIDMET is available from http://www.climatologylab.org/gridmet.html. Wildland-urban interface maps are available from http://silvis.forest.wisc.edu/data/wui-change/.

Field-specific reporting

Please select the one below that is the best fit for your research. If you are not sure, read the appropriate sections before making your selection.

☐ Life sciences ☐ Behavioural & social sciences ☒ Ecological, evolutionary & environmental sciences

For a reference copy of the document with all sections, see nature.com/documents/nr-reporting-summary-flat.pdf

Ecological, evolutionary & environmental sciences study design

All studies must disclose on these points even when the disclosure is negative.

Study description	This study estimates a new plant-functional trait called plant-water sensitivity. The trait quantifies the sensitivity of live fuel moisture content to climate-derived moisture balance. The trait is estimated using a regression of sattelite data.
Research sample	The sample consists of all vegetated pixels in the 12-most western states of USA.
Sampling strategy	Data not sampled. All data is used.
Data collection	Data was collected by two sattelite-driven methods. Live fuel moisture content data was used from another study. It was estimated from Sentinel-1 microwave backscatter and Landsat-8 reflectance. Climate-derived moisture balance (dead fuel moisture content) was also used from another study (GRIDMET). It was estimated from a water-balance model with PRISM (https://prism.oregonstate.edu/) as a forcing variable.
Timing and spatial scale	Spatial scale= 4 km (resolution of GRIDMET data)<br>Timing: 2016 - 2020
Data exclusions	Pixels which were covered for more than 50% of the area by open surface water, or developed land (buildings, etc.) were excluded.
Reproducibility	Only statistical experiments were performed in this study. No sampling is used. Reproducibility is supported by making the code available. Executing the scripts will reproduce the figures.
Randomization	No grouping was performed.
Blinding	Not applicable. Only environmental data was used.

Did the study involve field work? ☐ Yes ☒ No

Reporting for specific materials, systems and methods

We require information from authors about some types of materials, experimental systems and methods used in many studies. Here, indicate whether each material, system or method listed is relevant to your study. If you are not sure if a list item applies to your research, read the appropriate section before selecting a response.

Materials & experimental systems

n/a	Involved in the study	
☒	☐	Antibodies
☒	☐	Eukaryotic cell lines
☒	☐	Palaeontology and archaeology
☒	☐	Animals and other organisms
☒	☐	Human research participants
☒	☐	Clinical data
☒	☐	Dual use research of concern

Methods

n/a	Involved in the study	
☒	☐	ChIP-seq
☒	☐	Flow cytometry
☒	☐	MRI-based neuroimaging
