# Supplementary material for: Plant-water sensitivity regulates wildfire vulnerability
Source: Nat Ecol Evol. 2022 Feb 7;6(3):332–9. doi: 10.1038/s41559-021-01654-2 (PMC8913365; doi:10.1038/s41559-021-01654-2)
Supplement: Supplementary file 3 — Peer Review Information [file 41559_2021_1654_MOESM3_ESM.pdf]

nature portfolio

Peer Review Information

Journal: Nature Ecology & Evolution
Manuscript Title: Plant-Water Sensitivity Regulates Wildfire Vulnerability
Corresponding author name(s): Krishna Rao

Reviewer Comments & Decisions:

Decision Letter, initial version:

18th October 2021

*Please ensure you delete the link to your author homepage in this e-mail if you wish to forward it to your co-authors.

Dear Mr Rao,

Your manuscript entitled "Plant-Water Sensitivity Regulates Wildfire Vulnerability" has now been seen by two reviewers, whose comments are attached. The reviewers have raised a number of concerns which will need to be addressed before we can offer publication in Nature Ecology & Evolution. We will therefore need to see your responses to the criticisms raised and to some editorial concerns, along with a revised manuscript, before we can reach a final decision regarding publication.

We therefore invite you to revise your manuscript taking into account all reviewer and editor comments. Please highlight all changes in the manuscript text file.

We are committed to providing a fair and constructive peer-review process. Do not hesitate to contact us if there are specific requests from the reviewers that you believe are technically impossible or unlikely to yield a meaningful outcome.

When revising your manuscript:

* Include a “Response to reviewers” document detailing, point-by-point, how you addressed each reviewer comment. If no action was taken to address a point, you must provide a compelling argument. This response will be sent back to the reviewers along with the revised manuscript.

* If you have not done so already please begin to revise your manuscript so that it conforms to our Article format instructions at http://www.nature.com/natecolevol/info/final-submission. Refer also to any guidelines provided in this letter.

* Include a revised version of any required reporting checklist. It will be available to referees (and, potentially, statisticians) to aid in their evaluation if the manuscript goes back for peer review. A revised checklist is essential for re-review of the paper.

Please use the link below to submit your revised manuscript and related files:

Open Access This file is licensed under a Creative Commons Attribution 4.0 International License, which permits use, sharing, adaptation, distribution and reproduction in any medium or format, as long as you give appropriate credit to the original author(s) and the source, provide a link to the Creative Commons license, and indicate if changes were made. In the cases where the authors are anonymous, such as is the case for the reports of anonymous peer reviewers, author attribution should be to 'Anonymous Referee' followed by a clear attribution to the source work. The images or other third party material in this file are included in the article’s Creative Commons license, unless indicated otherwise in a credit line to the material. If material is not included in the article’s Creative Commons license and your intended use is not permitted by statutory regulation or exceeds the permitted use, you will need to obtain permission directly from the copyright holder. To view a copy of this license, visit http://creativecommons.org/licenses/by/4.0/.

[REDACTED]

Note: This URL links to your confidential home page and associated information about manuscripts you may have submitted, or that you are reviewing for us. If you wish to forward this email to co-authors, please delete the link to your homepage.

We hope to receive your revised manuscript within four to eight weeks. If you cannot send it within this time, please let us know. We will be happy to consider your revision so long as nothing similar has been accepted for publication at Nature Ecology & Evolution or published elsewhere.

Nature Ecology & Evolution is committed to improving transparency in authorship. As part of our efforts in this direction, we are now requesting that all authors identified as 'corresponding author' on published papers create and link their Open Researcher and Contributor Identifier (ORCID) with their account on the Manuscript Tracking System (MTS), prior to acceptance. ORCID helps the scientific community achieve unambiguous attribution of all scholarly contributions. You can create and link your ORCID from the home page of the MTS by clicking on 'Modify my Springer Nature account'. For more information please visit please visit www.springernature.com/orcid.

Please do not hesitate to contact me if you have any questions or would like to discuss these revisions further.

We look forward to seeing the revised manuscript and thank you for the opportunity to review your work.

[REDACTED]

Reviewer expertise:

Reviewer #1:

Reviewer #2:

Reviewer #3:

Reviewers' comments:

Reviewer #1 (Remarks to the Author):

This study investigates the link between fire-climate relationships and plant-water sensitivity (PWS), as estimated from remotely sensed products, in the western USA. As a secondary analysis, the authors intersect PWS with recent changes in the wildland-urban interface. The study is interesting,

Open Access This file is licensed under a Creative Commons Attribution 4.0 International License, which permits use, sharing, adaptation, distribution and reproduction in any medium or format, as long as you give appropriate credit to the original author(s) and the source, provide a link to the Creative Commons license, and indicate if changes were made. In the cases where the authors are anonymous, such as is the case for the reports of anonymous peer reviewers, author attribution should be to 'Anonymous Referee' followed by a clear attribution to the source work. The images or other third party material in this file are included in the article's Creative Commons license, unless indicated otherwise in a credit line to the material. If material is not included in the article's Creative Commons license and your intended use is not permitted by statutory regulation or exceeds the permitted use, you will need to obtain permission directly from the copyright holder. To view a copy of this license, visit http://creativecommons.org/licenses/by/4.0/.

original, and fits within the scope of the journal.

Much of this study hinges on the accuracy and relevance of remotely sensed products (ie, to estimate LFMC and DFMC). I realize that the methods for producing these estimates have been published, but I feel like most readers would like to know more about them. What is LFMC measuring, specifically? How accurate are the measures of LFMC? Another sentence or two would help.

I wondered if some confounding variables affected the reported relationships driven by PWS. The analyses provided in the supplementary material mostly convinced me that this was likely not the case. However, it seems possible that subtle confounding factors exert an influence on LFMC estimates. For instance, is plant size or morphology affecting LFMC estimates? Some authors have argued that plant structure should be considered in addition to fuel moisture to properly evaluate the effect of (Fernandes and Cruz 2012). This may be a non-issue if LFMC is measuring only moisture content, but it may be prudent to temper some statements about the importance of fuel moisture and eco-physiological processes.

Reading the introduction, I was under the impression that some analyses would be conducted by vegetation type. Although I realize this is beyond the scope of this study, establishing a stronger link between PWS and plant type could further help disentangle the factors driving PWS (as the authors suggest). With respect to the lack of correlation between fire-climate sensitivity and PWS in grassland (Fig. S4), I accept the authors interpretation. However, there may be other factors at play. Fuel moisture in graminoids (especially dead ones) is extremely responsive to atmospheric change. As such, the 15-day increments for LFMC may wash out some of the variation to which grasslands may be sensitive.

Specific comments

Line 45-46--I doubt that this statement applies to all parts of the western US. The WUI is also close to fire-suppression resources and, as such, benefit from greater protection that distant wildland, at least in some areas.

Line 59--As phrased, it is implied that the variation in fuel flammability is also three fold.

Line 240--Phenological stage or degree of grass curing could be added to this list.

Line 311--I am not a specialist of constrained regressions, but it unclear why constraining the coefficients to be non-negative will avoid overfitting.

Line 350--I could not find the span of years considered in the analysis.

Line 358--I do not know this product, but it would be nice to (in Fig. S4?) to have an idea of how accurate and up to date it is.

Fig. S2. The missing wedge of data (presumably from SAR coverage?) should be acknowledge

Open Access This file is licensed under a Creative Commons Attribution 4.0 International License, which permits use, sharing, adaptation, distribution and reproduction in any medium or format, as long as you give appropriate credit to the original author(s) and the source, provide a link to the Creative Commons license, and indicate if changes were made. In the cases where the authors are anonymous, such as is the case for the reports of anonymous peer reviewers, author attribution should be to 'Anonymous Referee' followed by a clear attribution to the source work. The images or other third party material in this file are included in the article's Creative Commons license, unless indicated otherwise in a credit line to the material. If material is not included in the article's Creative Commons license and your intended use is not permitted by statutory regulation or exceeds the permitted use, you will need to obtain permission directly from the copyright holder. To view a copy of this license, visit http://creativecommons.org/licenses/by/4.0/.

somewhere.

References

Fernandes, P.M. and Cruz, M.G., 2012. Plant flammability experiments offer limited insight into vegetation–fire dynamics interactions. New Phytologist, 194(3), pp.606-609.

Reviewer #2 (Remarks to the Author):

The manuscript by Rao et al. investigates whether the sensitivity of burned areas to climate in the western US is mediated by plant response to drought. Plant response to drought is assessed as the sensitivity of ecosystem moisture content to small variations in water balance over several drought seasons. Moisture content is assessed with a recently developed methodology based on remote sensing. The main results show that the sensitivity of vegetation moisture content to drought (named plant water sensitivity; PWS) regulates the effect of atmospheric aridity (VPD) on burned area. Some regions that combine with high PWS and where VPD increased faster than average. Finally, the authors show that the population in the WUI grew more rapidly in regions with high PWS. Altogether these results suggest that regions with PWS are more vulnerable to a climate-induced increase in VPD and that the increase in wildfire risk observed in the WUI would not be only due to increased ignitions but also to the higher vulnerability of the vegetation to fire in these regions.

The manuscript is well-written and the figures are clear. The results are important with exciting implications. Fuel-climate interactions, despite their importance for our broad understanding of wildfire regimes, are not well understood and seldom documented at large scales. In most cases, fire-climate relationships are established for different ecoregions or administrative regions, a method that offers only very limited insights into the mechanistic drivers of fire-climate interactions. Furthermore, the impact of fuel moisture on fire activity is rarely directly considered. This manuscript, therefore, fills several scientific gaps by showing that PWS mediates the effect of climate on burned area. This is all the more important because identifying the spatial drivers of BA is not straightforward. This is due to the fact that fire activity is driven by multiple drivers and is also very stochastic.

Despite these strengths, I have a number of recommendations and comments that I would like to be addressed before I feel comfortable recommending this manuscript for publication. My mains concern is that I found that it falls short when it comes to analyzing and understanding the patterns shown in figure 1 (which is the core of the study). I see two main reasons for this.

The first reason is that I am still wondering what are the factors explaining PWS. The authors claim that hydraulic traits could drive PWS (L56-L57, L63-L64, L69-70) but no proofs are given to confirm this intuition. Note that I appreciated the efforts made by the authors to discard a number of potential factors that might covary with PWS and affect the methodology (L111-126). However, this does not prove that hydraulic traits actually explain PWS. Relationships between LFMC and climate are complex and affected by multiple factors, including hydraulic traits, stand characteristics but also climate conditions. For instance, LFMC variations are expected to be less important when drought is higher

Open Access This file is licensed under a Creative Commons Attribution 4.0 International License, which permits use, sharing, adaptation, distribution and reproduction in any medium or format, as long as you give appropriate credit to the original author(s) and the source, provide a link to the Creative Commons license, and indicate if changes were made. In the cases where the authors are anonymous, such as is the case for the reports of anonymous peer reviewers, author attribution should be to 'Anonymous Referee' followed by a clear attribution to the source work. The images or other third party material in this file are included in the article's Creative Commons license, unless indicated otherwise in a credit line to the material. If material is not included in the article's Creative Commons license and your intended use is not permitted by statutory regulation or exceeds the permitted use, you will need to obtain permission directly from the copyright holder. To view a copy of this license, visit http://creativecommons.org/licenses/by/4.0/.

(due to high VPD or/and low soil moisture) because of stomatal closure. It is therefore difficult to foresee what are the main important factors driving PWS. I, therefore, encourage the authors to explore these issues. This could be done by comparing PWS to a series of potential co-variables, including vegetation type, hydraulics traits databases, or soil characteristics. This will require additional analyses but I think that this is a necessary step without which a part of the demonstration is missing.

The second reason is that I found that the methodology used to demonstrate the relationship between plant water sensitivity and wildfire vulnerability is not straightforward and this raises a number of issues regarding the meaning and interpretation of the results of Figure 1. Surprisingly, I found no specific discussion in the manuscript on why we should expect that the relationship between dBA/DVP is higher for higher PWS. Instead, potential explanations for these results are scattered throughout the text, suggesting that this might be explained by the impact of fuel moisture on fire behavior (e.g., L126) although it is not very clear if the authors refer to fuel moisture or Live fuel moisture. Demonstrating the impact of LFMC on fire behavior is beyond the scope of this manuscript as it would require analyses that specifically relate LFMC estimation with proxies of fire intensity (such as fire severity. However, I think it is important to clearly discuss the main hypotheses that could explain the results shown in Figure 1, notably because dBA/dVPD could be driven by other factors than LFMC. DFMC, for instance, is generally thought to be directly linked to VPD as well as an important driver of fire occurrence. Besides, the literature that relates live fuel moisture content to BA and fire behavior, is not mentioned. I think It might help discuss these results (e.g. Dennison and Moritz 2009, Nolan et al. 2016, Pimont et al. 2019)

The rest of the paper is less controversial. However, one key result is that human exposure to wildfire risk grew faster in high areas with high plant water sensitivity but this conclusion is not confirmed by analyses of BA trends. For instance, I would have expected to see analyses showing that for a similar VPD increase, burned area increases more in regions where vegetation moisture shows greater sensitivity to water limitation. But this does not appear in the manuscript. Any reason for this?

References

Dennison, P. E., & Moritz, M. A. (2009). Critical live fuel moisture in chaparral ecosystems: a threshold for fire activity and its relationship to antecedent precipitation. International Journal of Wildland Fire, 18(8), 1021-1027.

Nolan, R. H., Boer, M. M., Resco de Dios, V., Caccamo, G., & Bradstock, R. A. (2016). Large-scale, dynamic transformations in fuel moisture drive wildfire activity across southeastern Australia. Geophysical Research Letters, 43(9), 4229-4238.

Pimont, F., Ruffault, J., Martin-StPaul, N. K., & Dupuy, J. L. (2019). A cautionary note regarding the use of cumulative burnt areas for the determination of fire danger index breakpoints. International journal of wildland fire, 28(3), 254-258.

Open Access This file is licensed under a Creative Commons Attribution 4.0 International License, which permits use, sharing, adaptation, distribution and reproduction in any medium or format, as long as you give appropriate credit to the original author(s) and the source, provide a link to the Creative Commons license, and indicate if changes were made. In the cases where the authors are anonymous, such as is the case for the reports of anonymous peer reviewers, author attribution should be to 'Anonymous Referee' followed by a clear attribution to the source work. The images or other third party material in this file are included in the article's Creative Commons license, unless indicated otherwise in a credit line to the material. If material is not included in the article's Creative Commons license and your intended use is not permitted by statutory regulation or exceeds the permitted use, you will need to obtain permission directly from the copyright holder. To view a copy of this license, visit http://creativecommons.org/licenses/by/4.0/.

*******************END*******************

Author Rebuttal to Initial comments

Reviewer #1 (Remarks to the Author):

Reviewer: This study investigates the link between fire-climate relationships and plant-water sensitivity (PWS), as estimated from remotely sensed products, in the western USA. As a secondary analysis, the authors intersect PWS with recent changes in the wildland-urban interface. The study is interesting, original, and fits within the scope of the journal.

Response: We thank the reviewer for recognizing the originality and potential impact of our work.

Reviewer: Much of this study hinges on the accuracy and relevance of remotely sensed products (ie, to estimate LFMC and DFMC). I realize that the methods for producing these estimates have been published, but I feel like most readers would like to know more about them. What is LFMC measuring, specifically? How accurate are the measures of LFMC? Another sentence or two would help.

Response: The LFMC was estimated from microwave and optical remote-sensing using a deep learning model. The model was trained and validated on field-measured LFMC of leaves, needles, and thin branches. The field measurements for LFMC were obtained from the National Fuel Moisture Database (United States Forest Service 2018) which consists of geotagged and timestamped measurements. To clarify this, we add the following sentences to the manuscript (L402; Note: Line numbers henceforth refer to the document with changes highlighted):

> The LFMC dataset is trained on field-measured moisture content of live leaves, needles, and thin branches from the National Fuel Moisture Database (United States Forest Service 2018). The dataset has an overall R^2=0.63, RMSE=25%, and bias=-1.9%, with consistent performance across all land cover types.

Open Access This file is licensed under a Creative Commons Attribution 4.0 International License, which permits use, sharing, adaptation, distribution and reproduction in any medium or format, as long as you give appropriate credit to the original author(s) and the source, provide a link to the Creative Commons license, and indicate if changes were made. In the cases where the authors are anonymous, such as is the case for the reports of anonymous peer reviewers, author attribution should be to 'Anonymous Referee' followed by a clear attribution to the source work. The images or other third party material in this file are included in the article's Creative Commons license, unless indicated otherwise in a credit line to the material. If material is not included in the article's Creative Commons license and your intended use is not permitted by statutory regulation or exceeds the permitted use, you will need to obtain permission directly from the copyright holder. To view a copy of this license, visit http://creativecommons.org/licenses/by/4.0/.

Reviewer: I wondered if some confounding variables affected the reported relationships driven by PWS. The analyses provided in the supplementary material mostly convinced me that this was likely not the case. However, it seems possible that subtle confounding factors exert an influence on LFMC estimates. For instance, is plant size or morphology affecting LFMC estimates? Some authors have argued that plant structure should be considered in addition to fuel moisture to properly evaluate the effect of (Fernandes and Cruz 2012). This may be a non-issue if LFMC is measuring only moisture content, but it may be prudent to temper some statements about the importance of fuel moisture and eco-physiological processes.

Response: The LFMC specifically measures the moisture content of live leaves, needles, and branches. This is because the LFMC was estimated empirically (and rigorously cross-validated) with a deep learning model, the training data for which contains the raw LFMC values only. However, note that LFMC may still retain a non-zero correlation with plant size because of the known link between hydraulic traits and canopy height (McDowell *et al.* 2002; Novick *et al.* 2009).

To address this concern, we first calculate the Pearson correlation between PWS and canopy height (obtained from Simard *et al.* 2011) and find that it is very low (r = -0.04). Due to another set of concerns raised by reviewer 2 regarding the lack of explainable factors for PWS, we further investigate the spatial drivers of PWS using independent estimates of several different plant and soil hydraulic traits. We use a random forest regression with 13 variables (Table R1). The regression's fit (R^2 = 0.57) is sufficiently high to interpret its variable importance. We find that the canopy height is only 5% important (5% of the explained variance, Fig. R1 below). Since the importance is low, the chances of plant size confounding the effect of PWS on dBA/dVPD is also low.

Table R1: Variables used in random forest regression to evaluate spatial drivers of PWS.

Category	*Variable Name*	*Description*	*Source*
Vegetation	Canopy height	Height of canopy	(Simard et al. 2011)
	Xylem	Proportionality between water flux and the	(Liu et al. 2021)

Open Access This file is licensed under a Creative Commons Attribution 4.0 International License, which permits use, sharing, adaptation, distribution and reproduction in any medium or format, as long as you give appropriate credit to the original author(s) and the source, provide a link to the Creative Commons license, and indicate if changes were made. In the cases where the authors are anonymous, such as is the case for the reports of anonymous peer reviewers, author attribution should be to 'Anonymous Referee' followed by a clear attribution to the source work. The images or other third party material in this file are included in the article's Creative Commons license, unless indicated otherwise in a credit line to the material. If material is not included in the article's Creative Commons license and your intended use is not permitted by statutory regulation or exceeds the permitted use, you will need to obtain permission directly from the copyright holder. To view a copy of this license, visit http://creativecommons.org/licenses/by/4.0/.

	capacitance	corresponding change in xylem potential	
	g_1	Slope parameter in (Medlyn *et al.* 2011) which is inversely proportional to square root of water use efficiency. This is a stomatal trait.	(Liu et al. 2021)
	Max. xylem conductance	Maximum xylem conductance	(Liu et al. 2021)
	Hydraulic functional type	Derived from K-means clustering of plant traits	(Liu et al. 2021)
	ψ_{50}	Xylem water potential at 50% loss of xylem conductivity	(Liu et al. 2021)
	Isohydricity	Level of stomatal and xylem regulation	(Konings & Gentine 2017)
	Rooting depth	Maximum rooting depth	(Fan et al. 2017)
Soil	K_s	Average saturated hydraulic conductivity	(Montzka et al. 2017)
	Porosity	Average saturated volumetric water content of soil (soil porosity)	(Montzka et al. 2017)
	Silt fraction	Fraction of silt content in soil	(Liu et al. 2014)
	Sand fraction	Fraction of sand content in soil	(Liu et al. 2014)
	Clay fraction	Fraction of clay content in soil	(Liu et al. 2014)

Open Access This file is licensed under a Creative Commons Attribution 4.0 International License, which permits use, sharing, adaptation, distribution and reproduction in any medium or format, as long as you give appropriate credit to the original author(s) and the source, provide a link to the Creative Commons license, and indicate if changes were made. In the cases where the authors are anonymous, such as is the case for the reports of anonymous peer reviewers, author attribution should be to 'Anonymous Referee' followed by a clear attribution to the source work. The images or other third party material in this file are included in the article's Creative Commons license, unless indicated otherwise in a credit line to the material. If material is not included in the article's Creative Commons license and your intended use is not permitted by statutory regulation or exceeds the permitted use, you will need to obtain permission directly from the copyright holder. To view a copy of this license, visit http://creativecommons.org/licenses/by/4.0/.

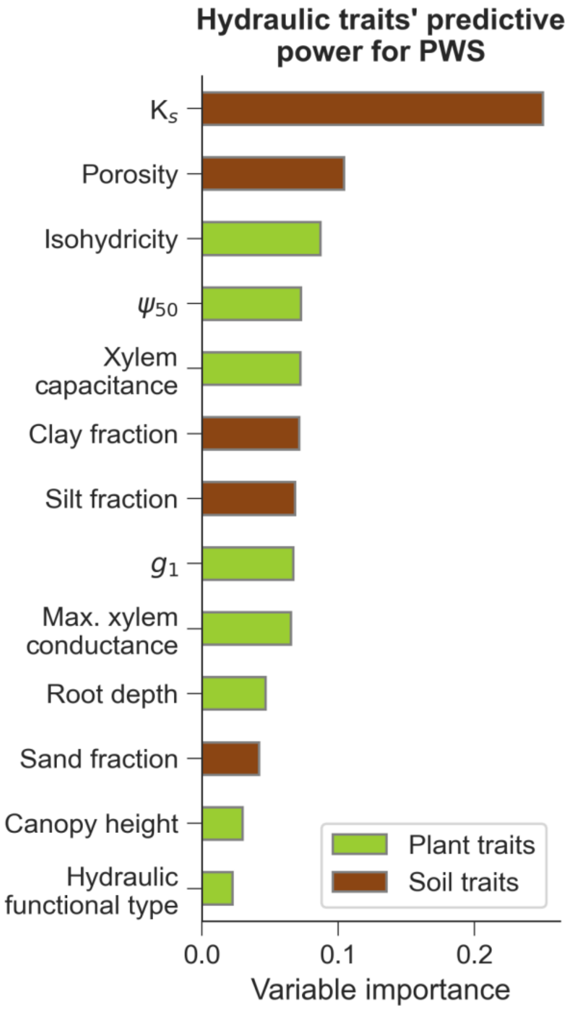

Fig. R1: Normalized variable importance from random forest regression to estimate PWS.

Based on the new results, we now explain in the manuscript that PWS is linked to many plant and soil hydraulic traits in L165:

> The strong link between PWS and $\frac{d\ burned\ area}{d\ VPD}$ prompts the question, what factors affect PWS? Using a random forest model (see Methods), we find that 57% of the variance in PWS is explained by 13 plant and soil hydraulic traits (Fig. S9). The most important drivers of PWS are average saturated soil hydraulic conductivity (25% importance),

Open Access This file is licensed under a Creative Commons Attribution 4.0 International License, which permits use, sharing, adaptation, distribution and reproduction in any medium or format, as long as you give appropriate credit to the original author(s) and the source, provide a link to the Creative Commons license, and indicate if changes were made. In the cases where the authors are anonymous, such as is the case for the reports of anonymous peer reviewers, author attribution should be to 'Anonymous Referee' followed by a clear attribution to the source work. The images or other third party material in this file are included in the article's Creative Commons license, unless indicated otherwise in a credit line to the material. If material is not included in the article's Creative Commons license and your intended use is not permitted by statutory regulation or exceeds the permitted use, you will need to obtain permission directly from the copyright holder. To view a copy of this license, visit http://creativecommons.org/licenses/by/4.0/.

> average saturated volumetric water content (10% importance), and isohydricity—which describes xylem and stomatal regulation (9% importance; Fig. 2). In total, soil and plant traits contribute to 54% and 46% of the explained importance, respectively.

We also include additional results from the analysis which is explained in our response to the next reviewer comment below.

Reviewer: Reading the introduction, I was under the impression that some analyses would be conducted by vegetation type. Although I realize this is beyond the scope of this study, establishing a stronger link between PWS and plant type could further help disentangle the factors driving PWS (as the authors suggest).

Response: Fig. R1 shows the variable importance of several plant and soil factors that drive variation in PWS. We find that vegetation type as categorized by hydraulic function type (HFT) has the lowest feature importance. The HFT is an improved categorization of vegetation relative to plant functional type (PFT) that is specifically based on assimilated plant hydraulic traits (Liu *et al.* 2021), since hydraulic traits can have high variability within a given land cover type (Anderegg 2015). Despite their improved relevance to plant hydraulics, HFTs have very low explanatory power for PWS.

To explain this, we add the following sentence to the manuscript (new text in bold) L239:

> Global fire models parametrize vegetation controls on burned area using plant functional types (Hantson *et al.* 2016; Rabin *et al.* 2017). **However, the plant and soil hydraulic traits that control PWS (Fig. S2) are known to vary significantly within PFT types (e.g., Anderegg 2015; Konings & Gentine 2017). This suggests that basing vegetation-fire relationships on functional types may lead to large errors (Forkel *et al.* 2019).** The PWS metric is the first that, (i) directly quantifies vegetation-climate sensitivity, (ii) is shown to be related to burned area, and (iii) is scalable globally. The PWS metric thus offers a potential pathway to improve global vegetation-fire modeling.

Although we do not find a relationship between PWS and plant type, we do find significant relationships with other plant and soil hydraulic traits. This is now explained in L246:

Open Access This file is licensed under a Creative Commons Attribution 4.0 International License, which permits use, sharing, adaptation, distribution and reproduction in any medium or format, as long as you give appropriate credit to the original author(s) and the source, provide a link to the Creative Commons license, and indicate if changes were made. In the cases where the authors are anonymous, such as is the case for the reports of anonymous peer reviewers, author attribution should be to 'Anonymous Referee' followed by a clear attribution to the source work. The images or other third party material in this file are included in the article's Creative Commons license, unless indicated otherwise in a credit line to the material. If material is not included in the article's Creative Commons license and your intended use is not permitted by statutory regulation or exceeds the permitted use, you will need to obtain permission directly from the copyright holder. To view a copy of this license, visit http://creativecommons.org/licenses/by/4.0/.

Although PWS is not a widely recognized plant trait, we show that it is an indicator of whole-plant water relations. It combines several plant and soil hydraulic traits that affect LFMC (Fig. 2). Notably, soil hydraulic traits explain a slightly larger fraction (54% vs. 46%) of the variation in PWS than plant hydraulic traits do. This is to some extent expected; datasets of the explanatory variables have much coarser resolution than that of PWS, and because vegetation traits are more spatially heterogeneous than soil traits, this mismatch in scales will add noise and reduce the explanatory power of the plant hydraulic traits more than that of the soil hydraulic traits. Furthermore, because PWS is determined from LFMC, a property of the vegetation, even a co-variation with soil hydraulic traits merely reflects the way that plant-water uptake is affected by soil hydraulics. Thus, we argue that our interpretation of PWS as a metric of the water sensitivity of plants is appropriate despite the influence of soil hydraulic traits, as further discussed in Text S3.

We interpret the variable importance in more detail in Text S3:

Plant and soil hydraulic traits both influence PWS. Since PWS is derived from LFMC's sensitivity to lagged climate-derived moisture balance (see Methods), all hydraulic traits that affect LFMC are expected to influence PWS too. We broadly categorize all hydraulic traits as soil traits or plant traits (Table S1), but each trait affects plant moisture in different stages of the soil-plant continuum: from root access to soil water, to water transport through the xylem and leaves, and finally to transpiration. Our ability to identify the importance of the hydraulic traits belonging to each of the stages is limited by data quality. Thus, the relative importance of the variables used in the random forest regression is affected by at least three factors. First, accuracy of data on soil hydraulic traits is higher than plant hydraulic traits. Soil hydraulic traits are derived from intensively sampled in-situ measurements and sub-1-km scale data (Montzka *et al.* 2017). By contrast, most of the plant hydraulic traits we use are retrieved from data assimilation of satellite observables (Liu *et al.* 2021), which are sensitive to errors in the plant hydraulic model underlying the data assimilation, remote sensing retrieval algorithm errors, and uncertainties in the data assimilation methodology. The magnitude of these uncertainties is reflected in the considerable magnitude of the estimated uncertainty for these hydraulic traits (Liu et al., 2021). Second, there is a large mismatch in the spatial

Open Access This file is licensed under a Creative Commons Attribution 4.0 International License, which permits use, sharing, adaptation, distribution and reproduction in any medium or format, as long as you give appropriate credit to the original author(s) and the source, provide a link to the Creative Commons license, and indicate if changes were made. In the cases where the authors are anonymous, such as is the case for the reports of anonymous peer reviewers, author attribution should be to 'Anonymous Referee' followed by a clear attribution to the source work. The images or other third party material in this file are included in the article's Creative Commons license, unless indicated otherwise in a credit line to the material. If material is not included in the article's Creative Commons license and your intended use is not permitted by statutory regulation or exceeds the permitted use, you will need to obtain permission directly from the copyright holder. To view a copy of this license, visit http://creativecommons.org/licenses/by/4.0/.

resolution of the hydraulic traits and PWS. All hydraulic traits except for canopy height (300 m resolution, though derived from a ten-year old dataset) and root depth (1 km resolution) are available only at 25 km resolution, whereas PWS is available at 4 km resolution. The spatial mismatch may have adversely impacted the importance of plant hydraulic traits, since they are known to be extremely heterogenous (Anderegg 2015). By contrast, the effect of this mismatch should be smaller for soil hydraulic traits, which are generally more homogenous (Liu *et al.* 2014). Third, 43% of the variation in PWS is unaccounted for in our analysis. The unexplained variance might be due to other plant hydraulic traits which are not included in our analysis (Trugman *et al.* 2019). All three above-mentioned factors combined with the near-equal influence of soil and plant hydraulic traits on PWS (Fig. 2) suggests PWS represents whole-plant water relations and not just soil-water relations alone.

Finally, we explain the investigation into the spatial drivers of PWS in a new Methods section L436:

To investigate what factors influence PWS, we regress the PWS map against eight plant hydraulic traits and five soil hydraulic traits using an ensemble approach. For plant traits, we use canopy height from 2005 from (Simard *et al.* 2011) and several hydraulic traits including xylem capacitance, a stomatal conductance slope parameter which is inversely proportional to marginal water use efficiency (g_1), maximum xylem conductance, the xylem water potential at 50% loss of conductance (ψ_{50}), and a hydraulic functional type derived from K-means clustering of these plant hydraulic traits determined from (41). We also include isohydricity (a stomatal regulation trait) from (Konings & Gentine 2017), and maximum rooting depth from (Fan *et al.* 2017). For soil traits, we use average saturated hydraulic conductivity (K_s), soil porosity from (Montzka *et al.* 2017) and fraction of soil, silt, and clay from (Liu *et al.* 2014) (Table S1). We rescale all traits to 4-km resolution using nearest neighbor interpolation since all traits (except for canopy height and rooting depth) have a coarse resolution of 25 km.

We use the rescaled traits as explanatory variables in a random forest regression to assess the importance of each trait. We ignore all time-varying factors in our analysis, and use static maps of all traits to explain the variance in the static map of PWS. We use 3-fold

Open Access This file is licensed under a Creative Commons Attribution 4.0 International License, which permits use, sharing, adaptation, distribution and reproduction in any medium or format, as long as you give appropriate credit to the original author(s) and the source, provide a link to the Creative Commons license, and indicate if changes were made. In the cases where the authors are anonymous, such as is the case for the reports of anonymous peer reviewers, author attribution should be to 'Anonymous Referee' followed by a clear attribution to the source work. The images or other third party material in this file are included in the article's Creative Commons license, unless indicated otherwise in a credit line to the material. If material is not included in the article's Creative Commons license and your intended use is not permitted by statutory regulation or exceeds the permitted use, you will need to obtain permission directly from the copyright holder. To view a copy of this license, visit http://creativecommons.org/licenses/by/4.0/.

> cross-validation to evaluate strength of fit (Fig. S9). To compute variable importance, we measure the average decrease in node impurity due to splitting at each variable and normalize the output (Fig. 2). For the random forest ensembles, we choose a minimum of six samples per terminal node, a minimum of 5 x 10^{-6} reduction in node impurity at each split, a minimum of two samples split per node, bootstrapped samples during node splits, and 50 trees within each forest. We use scikit-learn for the random forest regression (Pedregosa *et al*. 2011).

Reviewer: With respect to the lack of correlation between fire-climate sensitivity and PWS in grassland (Fig. S4), I accept the authors interpretation. However, there may be other factors at play. Fuel moisture in graminoids (especially dead ones) is extremely responsive to atmospheric change. As such, the 15-day increments for LFMC may wash out some of the variation to which grasslands may be sensitive.

Response: Yes, the very low response times to water limitation in grasslands may limit our ability to investigate the effect of PWS on dBA/dVPD. We add the following clause (new text in bold) to a pre-existing sentence on L282:

> Since our data show that VPD played an insignificant role in governing burned area variability for grasslands (Fig. S4), **and since the LFMC data's temporal resolution of 15 days may be insufficient to capture rapid responses to water limitation in grasslands,** we are unable to quantify the regulating role of PWS on the response of burned area to VPD in grassland ecosystems.

Specific comments

Reviewer: Line 45-46--I doubt that this statement applies to all parts of the western US. The WUI is also close to fire-suppression resources and, as such, benefit from greater protection that distant wildland, at least in some areas.

Response: The sentence in question is:

Open Access This file is licensed under a Creative Commons Attribution 4.0 International License, which permits use, sharing, adaptation, distribution and reproduction in any medium or format, as long as you give appropriate credit to the original author(s) and the source, provide a link to the Creative Commons license, and indicate if changes were made. In the cases where the authors are anonymous, such as is the case for the reports of anonymous peer reviewers, author attribution should be to 'Anonymous Referee' followed by a clear attribution to the source work. The images or other third party material in this file are included in the article's Creative Commons license, unless indicated otherwise in a credit line to the material. If material is not included in the article's Creative Commons license and your intended use is not permitted by statutory regulation or exceeds the permitted use, you will need to obtain permission directly from the copyright holder. To view a copy of this license, visit http://creativecommons.org/licenses/by/4.0/.

> The WUI is burdened by greater fire hazard because human-caused ignitions are more frequent and there are greater risks to human lives and structures (Syphard *et al.* 2017; Mietkiewicz *et al.* 2020).

The WUI areas might have access to better suppression resources than distant wildlands, but evidence from literature suggests it has not amounted to less hazard in WUI areas. According to an analysis conducted by Mietkiewicz *et al.* (2020), WUI fires are almost exclusively human-caused, and 97% of homes within the WUI in USA were threatened by human-caused fires between 1992 to 2015. In the same period, the number of homes threatened by human-caused ignitions was 59 million in the WUI and 8 million in wildlands (see Table 1 in Mietkiewicz *et al.* (2020)). Similar findings are supported by Syphard *et al.* (2017) and Balch *et al.* (2017). Furthermore, relative to the more densely populated suburbs and urban areas (where more people live than in distant wildlands), fire suppression resources are likely to be worse in the more sparsely populated WUI. Nevertheless, some exceptions within the WUI areas might exist. To acknowledge that fact, we modify the sentence in question (edit in bold) L45:

> **Most of** the WUI is burdened by greater fire hazard because human-caused ignitions are more frequent and there are greater risks to human lives and structures (Balch *et al.* 2017; Syphard *et al.* 2017; Mietkiewicz *et al.* 2020).

<u>Reviewer:</u> Line 59--As phrased, it is implied that the variation in fuel flammability is also three fold.

<u>Response:</u> The original sentence read:

> Specifically, plant hydraulic traits can cause up to three-fold variation in fuel moisture and, thus, fuel flammability (Scarff *et al.* 2021).

We did not intend to claim that plant hydraulic traits can cause three-fold variation in fuel flammability. We acknowledge that the relationship between LFMC and flammability is non-linear. We modify the sentence to (L61):

> Specifically, plant hydraulic traits can cause up to three-fold variation in fuel moisture and, thus **affect** fuel flammability (Scarff *et al.* 2021).

Open Access This file is licensed under a Creative Commons Attribution 4.0 International License, which permits use, sharing, adaptation, distribution and reproduction in any medium or format, as long as you give appropriate credit to the original author(s) and the source, provide a link to the Creative Commons license, and indicate if changes were made. In the cases where the authors are anonymous, such as is the case for the reports of anonymous peer reviewers, author attribution should be to 'Anonymous Referee' followed by a clear attribution to the source work. The images or other third party material in this file are included in the article's Creative Commons license, unless indicated otherwise in a credit line to the material. If material is not included in the article's Creative Commons license and your intended use is not permitted by statutory regulation or exceeds the permitted use, you will need to obtain permission directly from the copyright holder. To view a copy of this license, visit http://creativecommons.org/licenses/by/4.0/.

Reviewer: Line 240--Phenological stage or degree of grass curing could be added to this list.

Response: We add "phenological stage" to our list. The degree of grass curing is highly linked to LFMC (Yebra *et al.* 2007). Since the LFMC is used to estimate PWS, we do not mention grass curing separately among the factors affecting the relationship between dBA/dVPD and PWS. The new sentence reads L279:

> In grasslands, which are largely located in the Great Plains, the low slope between annual burned area and VPD suggests that other factors such as availability of fuels, ignitions, and strong winds, **and phenological stage** may have more dominant influences on burned area (Knapp 1998; Keeley & Syphard 2016).

Line 311--I am not a specialist of constrained regressions, but it unclear why constraining the coefficients to be non-negative will avoid overfitting.

Response: Constraining the regression coefficients to be non-negative will avoid overfitting because an increase in climate-derived moisture balance anomaly will not be allowed to negatively affect an associated LFMC anomaly, as this would be unrealistic and would only arise due to over-fitting. This explanation was originally part of a parenthetical sentence which could have caused confusion. We edit the sentence so that the explanation is clearer L359:

> Unlike previous studies that use an unconstrained regression (García *et al.* 2008), we constrain the regression coefficients to be non-negative. We thus do not allow an increase in climate-derived moisture balance anomaly to negatively affect an associated LFMC anomaly, which would be unphysical.

Line 350--I could not find the span of years considered in the analysis.

Response: We add the following sentence to the Methods (L387):

> We use data from 2016 to 2020 to estimate PWS.

Open Access This file is licensed under a Creative Commons Attribution 4.0 International License, which permits use, sharing, adaptation, distribution and reproduction in any medium or format, as long as you give appropriate credit to the original author(s) and the source, provide a link to the Creative Commons license, and indicate if changes were made. In the cases where the authors are anonymous, such as is the case for the reports of anonymous peer reviewers, author attribution should be to 'Anonymous Referee' followed by a clear attribution to the source work. The images or other third party material in this file are included in the article's Creative Commons license, unless indicated otherwise in a credit line to the material. If material is not included in the article's Creative Commons license and your intended use is not permitted by statutory regulation or exceeds the permitted use, you will need to obtain permission directly from the copyright holder. To view a copy of this license, visit http://creativecommons.org/licenses/by/4.0/.

Line 358--I do not know this product, but it would be nice to (in Fig. S4?) to have an idea of how accurate and up to date it is.

Response: The original sentence read:

> Vegetated areas include regions with trees, shrubs, and grasslands obtained from GlobCover (Arino *et al.* 2010).

Upon careful inspection, we find that this was a mistake. We do not use GlobCover for our analysis. We use the National Land Cover Database (NLCD) version 2016 for identifying vegetated areas (as correctly noted in the caption of Fig. S4). We apologize for our mistake. The new sentence reads L413:

> Vegetated areas include regions with trees, shrubs, and grasslands obtained from the National Land Cover Database version 2016 (Homer *et al.* 2011)

We use the 2016 version of NLCD because that is the latest available. The NLCD uses Landsat data to estimate land cover. It has 82% accuracy. We update Fig S4 and its caption to reflect the same:

> Land cover map for 2016 from the National Land Cover Database (Homer *et al.* 2011) shown in the bottom row. The land cover map is based on inputs from Landsat data. The dataset has been validated extensively in the USA with an overall area-weighted accuracy of 82% (Homer & Fry 2012).

Reviewer: Fig. S2. The missing wedge of data (presumably from SAR coverage?) should be acknowledge somewhere.

Response: Yes, the large grey streak in the PWS map is due to missing SAR coverage. The PWS is also missing in cases where more than 50% of LFMC data record is unavailable due to snow and cloud cover. We add the following sentence to Fig. S2's caption:

> Grey patches in the PWS map indicate areas where PWS is unavailable due to missing Sentinel-1 data, snow cover, or cloud cover (see Methods).

And edit the following sentence in the Methods section (edits in bold) L391:

Open Access This file is licensed under a Creative Commons Attribution 4.0 International License, which permits use, sharing, adaptation, distribution and reproduction in any medium or format, as long as you give appropriate credit to the original author(s) and the source, provide a link to the Creative Commons license, and indicate if changes were made. In the cases where the authors are anonymous, such as is the case for the reports of anonymous peer reviewers, author attribution should be to 'Anonymous Referee' followed by a clear attribution to the source work. The images or other third party material in this file are included in the article's Creative Commons license, unless indicated otherwise in a credit line to the material. If material is not included in the article's Creative Commons license and your intended use is not permitted by statutory regulation or exceeds the permitted use, you will need to obtain permission directly from the copyright holder. To view a copy of this license, visit http://creativecommons.org/licenses/by/4.0/.

The PWS is not computed for the **9.5%** of pixels where more than 50% of the LFMC temporal record is missing due to cloud or snow cover, **or where synthetic aperture radar data is absent.**

Open Access This file is licensed under a Creative Commons Attribution 4.0 International License, which permits use, sharing, adaptation, distribution and reproduction in any medium or format, as long as you give appropriate credit to the original author(s) and the source, provide a link to the Creative Commons license, and indicate if changes were made. In the cases where the authors are anonymous, such as is the case for the reports of anonymous peer reviewers, author attribution should be to 'Anonymous Referee' followed by a clear attribution to the source work. The images or other third party material in this file are included in the article's Creative Commons license, unless indicated otherwise in a credit line to the material. If material is not included in the article's Creative Commons license and your intended use is not permitted by statutory regulation or exceeds the permitted use, you will need to obtain permission directly from the copyright holder. To view a copy of this license, visit http://creativecommons.org/licenses/by/4.0/.

Reviewer #2 (Remarks to the Author):

Reviewer: The manuscript by Rao et al. investigates whether the sensitivity of burned areas to climate in the western US is mediated by plant response to drought. Plant response to drought is assessed as the sensitivity of ecosystem moisture content to small variations in water balance over several drought seasons. Moisture content is assessed with a recently developed methodology based on remote sensing. The main results show that the sensitivity of vegetation moisture content to drought (named plant water sensitivity; PWS) regulates the effect of atmospheric aridity (VPD) on burned area. Some regions that combine with high PWS and where VPD increased faster than average. Finally, the authors show that the population in the WUI grew more rapidly in regions with high PWS. Altogether these results suggest that regions with PWS are more vulnerable to a climate-induced increase in VPD and that the increase in wildfire risk observed in the WUI would not be only due to increased ignitions but also to the higher vulnerability of the vegetation to fire in these regions.

The manuscript is well-written and the figures are clear. The results are important with exciting implications. Fuel-climate interactions, despite their importance for our broad understanding of wildfire regimes, are not well understood and seldom documented at large scales. In most cases, fire-climate relationships are established for different ecoregions or administrative regions, a method that offers only very limited insights into the mechanistic drivers of fire-climate interactions. Furthermore, the impact of fuel moisture on fire activity is rarely directly considered. This manuscript, therefore, fills several scientific gaps by showing that PWS mediates the effect of climate on burned area. This is all the more important because identifying the spatial drivers of BA is not straightforward. This is due to the fact that fire activity is driven by multiple drivers and is also very stochastic.

Response: We thank the reviewer for recognizing the importance of our work.

Reviewer: Despite these strengths, I have a number of recommendations and comments that I would like to be addressed before I feel comfortable recommending this manuscript for publication. My mains concern is that I found that it falls short when it comes to analyzing and understanding the patterns shown in figure 1 (which is the core of the study). I see two main reasons for this.

Open Access This file is licensed under a Creative Commons Attribution 4.0 International License, which permits use, sharing, adaptation, distribution and reproduction in any medium or format, as long as you give appropriate credit to the original author(s) and the source, provide a link to the Creative Commons license, and indicate if changes were made. In the cases where the authors are anonymous, such as is the case for the reports of anonymous peer reviewers, author attribution should be to 'Anonymous Referee' followed by a clear attribution to the source work. The images or other third party material in this file are included in the article's Creative Commons license, unless indicated otherwise in a credit line to the material. If material is not included in the article's Creative Commons license and your intended use is not permitted by statutory regulation or exceeds the permitted use, you will need to obtain permission directly from the copyright holder. To view a copy of this license, visit http://creativecommons.org/licenses/by/4.0/.

The first reason is that I am still wondering what are the factors explaining PWS. The authors claim that hydraulic traits could drive PWS (L56-L57, L63-L64, L69-70) but no proofs are given to confirm this intuition. Note that I appreciated the efforts made by the authors to discard a number of potential factors that might covary with PWS and affect the methodology (L111-126). However, this does not prove that hydraulic traits actually explain PWS. Relationships between LFMC and climate are complex and affected by multiple factors, including hydraulic traits, stand characteristics but also climate conditions. For instance, LFMC variations are expected to be less important when drought is higher (due to high VPD or/and low soil moisture) because of stomatal closure. It is therefore difficult to foresee what are the main important factors driving PWS. I, therefore, encourage the authors to explore these issues. This could be done by comparing PWS to a series of potential co-variables, including vegetation type, hydraulics traits databases, or soil characteristics. This will require additional analyses but I think that this is a necessary step without which a part of the demonstration is missing.

Response: Based on similar concerns shared by both reviewers, we now investigate whether and to what extent plant and soil hydraulic traits drive the spatial variation in PWS. We do this using random forest regression because it allows easy interpretation of the importance of traits. We use eight plant hydraulic traits and five soil hydraulic traits to explain the variance in PWS. The traits along with their source are shown below in Table R2.

Table R2: Variables used in random forest regression to evaluate spatial drivers of PWS.

Category	*Variable Name*	*Description*	*Source*
Vegetation	Canopy height	Height of canopy	(Simard et al. 2011)
	Xylem capacitance	Proportionality between water flux and the corresponding change in xylem potential	(Liu et al. 2021)
	g_1	Slope parameter in (Medlyn *et al.* 2011) which is inversely proportional to square root of water use efficiency. This is a stomatal trait.	(Liu et al. 2021)
	Max. xylem conductance	Maximum xylem conductance	(Liu et al. 2021)

Open Access This file is licensed under a Creative Commons Attribution 4.0 International License, which permits use, sharing, adaptation, distribution and reproduction in any medium or format, as long as you give appropriate credit to the original author(s) and the source, provide a link to the Creative Commons license, and indicate if changes were made. In the cases where the authors are anonymous, such as is the case for the reports of anonymous peer reviewers, author attribution should be to 'Anonymous Referee' followed by a clear attribution to the source work. The images or other third party material in this file are included in the article's Creative Commons license, unless indicated otherwise in a credit line to the material. If material is not included in the article's Creative Commons license and your intended use is not permitted by statutory regulation or exceeds the permitted use, you will need to obtain permission directly from the copyright holder. To view a copy of this license, visit http://creativecommons.org/licenses/by/4.0/.

	Hydraulic functional type	Derived from K-means clustering of plant traits	(Liu et al. 2021)
	ψ_{50}	Xylem water potential at 50% loss of xylem conductivity	(Liu et al. 2021)
	Isohydricity	Level of stomatal and xylem regulation	(Konings & Gentine 2017)
	Rooting depth	Maximum rooting depth	(Fan et al. 2017)
Soil	K_s	Average saturated hydraulic conductivity	(Montzka et al. 2017)
	Porosity	Average saturated volumetric water content of soil (soil porosity)	(Montzka et al. 2017)
	Silt fraction	Fraction of silt content in soil	(Liu et al. 2014)
	Sand fraction	Fraction of sand content in soil	(Liu et al. 2014)
	Clay fraction	Fraction of clay content in soil	(Liu et al. 2014)

As seen from the table, we use a variety of traits from the soil-plant continuum that affect vegetation's ability to access water during water limitation, thus influencing PWS. These traits include:

1. Soil hydraulic traits: We use saturated hydraulic conductivity and volumetric water content at saturation because they control infiltration and the redistribution of water throughout and away from the rooting zone, thus potentially affecting PWS. We also use silt, sand, and clay fraction as potential factors. These traits affect the relationship between porosity and soil water potential.
2. Plant hydraulic traits:
    a. Below-ground: rooting depth

Open Access This file is licensed under a Creative Commons Attribution 4.0 International License, which permits use, sharing, adaptation, distribution and reproduction in any medium or format, as long as you give appropriate credit to the original author(s) and the source, provide a link to the Creative Commons license, and indicate if changes were made. In the cases where the authors are anonymous, such as is the case for the reports of anonymous peer reviewers, author attribution should be to 'Anonymous Referee' followed by a clear attribution to the source work. The images or other third party material in this file are included in the article's Creative Commons license, unless indicated otherwise in a credit line to the material. If material is not included in the article's Creative Commons license and your intended use is not permitted by statutory regulation or exceeds the permitted use, you will need to obtain permission directly from the copyright holder. To view a copy of this license, visit http://creativecommons.org/licenses/by/4.0/.

b. Xylem-level: Xylem water potential at 50% loss of xylem conductivity, xylem capacitance, xylem conductance, and canopy height

c. Leaf-level: Isohydricity, and a metric related to water-use efficiency.

When we train our random forest regression and use it to reconstruct PWS in a separate hold-out validation set, the above variables explain 57% of the variance in PWS (Fig. R2). The explained variance is likely suppressed by the coarse resolution of most of the traits data. With the exception of canopy height and rooting depth, all traits are available at 25 km resolution only (compared PWS resolution of 4 km). Thus, despite the low variance we believe the model is sufficiently accurate to be able to interpret the importance of variables towards its prediction. Furthermore, other plant hydraulic traits are uncertain. The rooting depth estimates for example, are the best available estimates of rooting depth at this scale and across the entire western US, but are derived from inverting a hydrologic model (Fan *et al.* 2017). All errors in the parameterization of stomatal closure and other factors simulated in the hydrologic models will therefore propagate to the rooting depth estimates and reduce the explained variability of the random forest regression. Similarly, the xylem level and several of the leaf-level estimates are also a product of data assimilation, with model errors adding noise (Liu *et al.* 2021). Lastly, all xylem and leaf-level datasets are derived from noisy remote sensing observations, and the isohydricity estimates rely on several other simplifying assumptions about the frequency and rapidity of pre-dawn equilibrium and the shape of plant pressure-volume curves (Konings & Gentine 2017). All in all, these factors cause us to interpret a 57% explained variance as reasonable, and sufficient for further interpretation of the relative importance of different variables in this model.

Open Access This file is licensed under a Creative Commons Attribution 4.0 International License, which permits use, sharing, adaptation, distribution and reproduction in any medium or format, as long as you give appropriate credit to the original author(s) and the source, provide a link to the Creative Commons license, and indicate if changes were made. In the cases where the authors are anonymous, such as is the case for the reports of anonymous peer reviewers, author attribution should be to 'Anonymous Referee' followed by a clear attribution to the source work. The images or other third party material in this file are included in the article's Creative Commons license, unless indicated otherwise in a credit line to the material. If material is not included in the article's Creative Commons license and your intended use is not permitted by statutory regulation or exceeds the permitted use, you will need to obtain permission directly from the copyright holder. To view a copy of this license, visit http://creativecommons.org/licenses/by/4.0/.

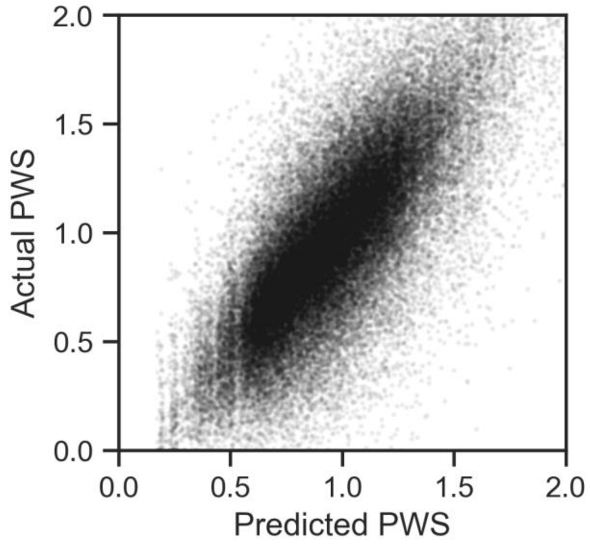

Fig. R2: Performance of the random forest regression on leave-out validation set. “Actual PWS” refers to the PWS used in the manuscript. “Predicted PWS” refers to the PWS from the random forest regression. R^2 for the fit is 0.57.

The variable importance from the random forest regression shows that saturated soil hydraulic conductivity (K_s) is by far the largest driver of PWS, followed by other soil and plant hydraulic traits (Fig. R3). The K_s has previously been shown to exert a dominant influence on root water uptake (Verhoef & Egea 2014). Isohydricity, a trait indicating the level of stomatal regulation, is the most important plant hydraulic trait. Hydraulic function type, an improved categorization scheme over plant function types, is the least important driver of PWS. Overall, soil hydraulic traits amount to 54% of the variable importance (cumulative length of all brown bars in Fig. R3), whereas plant traits contributed to 46% of the importance (cumulative length of green bars).

Open Access This file is licensed under a Creative Commons Attribution 4.0 International License, which permits use, sharing, adaptation, distribution and reproduction in any medium or format, as long as you give appropriate credit to the original author(s) and the source, provide a link to the Creative Commons license, and indicate if changes were made. In the cases where the authors are anonymous, such as is the case for the reports of anonymous peer reviewers, author attribution should be to 'Anonymous Referee' followed by a clear attribution to the source work. The images or other third party material in this file are included in the article's Creative Commons license, unless indicated otherwise in a credit line to the material. If material is not included in the article's Creative Commons license and your intended use is not permitted by statutory regulation or exceeds the permitted use, you will need to obtain permission directly from the copyright holder. To view a copy of this license, visit http://creativecommons.org/licenses/by/4.0/.

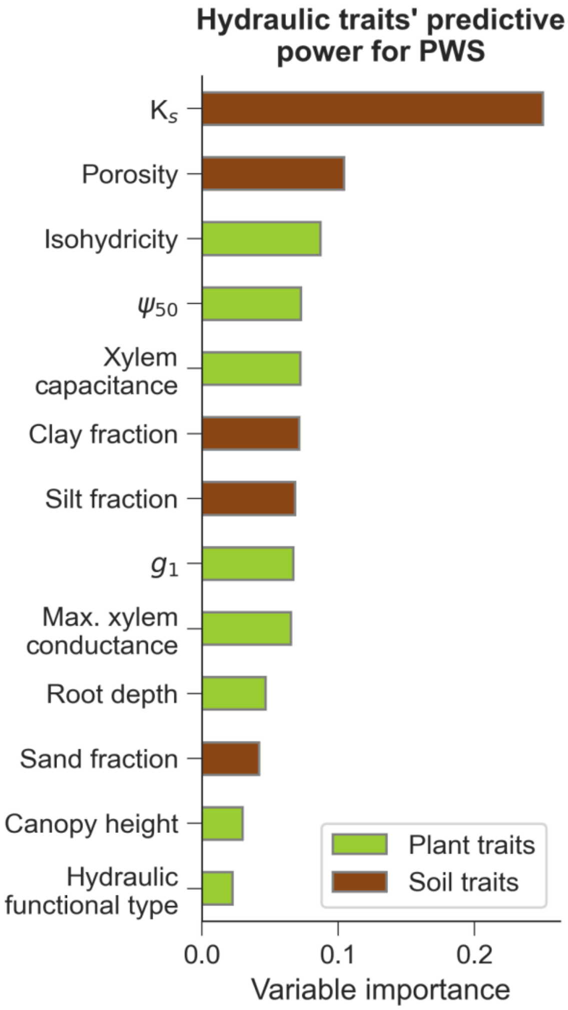

Fig. R3: Normalized variable importance from random forest regression to estimate PWS.

From this analysis we conclude that plant and soil hydraulic traits indeed drive most of the variation in PWS, although our ability to investigate the PWS drivers might be limited by the spatial resolution and noisy nature of the hydraulic traits themselves. In the case of plant hydraulic traits, the coarse spatial resolution is likely especially impactful, since plant traits can be extremely heterogeneous in space, often varying by many orders of magnitude among and sometimes even within species (Anderegg 2015). Furthermore, because the PWS is derived from an LFMC dataset, we note that the soil hydraulic traits’ importance likely reflect how soil

Open Access This file is licensed under a Creative Commons Attribution 4.0 International License, which permits use, sharing, adaptation, distribution and reproduction in any medium or format, as long as you give appropriate credit to the original author(s) and the source, provide a link to the Creative Commons license, and indicate if changes were made. In the cases where the authors are anonymous, such as is the case for the reports of anonymous peer reviewers, author attribution should be to 'Anonymous Referee' followed by a clear attribution to the source work. The images or other third party material in this file are included in the article’s Creative Commons license, unless indicated otherwise in a credit line to the material. If material is not included in the article’s Creative Commons license and your intended use is not permitted by statutory regulation or exceeds the permitted use, you will need to obtain permission directly from the copyright holder. To view a copy of this license, visit http://creativecommons.org/licenses/by/4.0/.

hydraulic traits affect plant water uptake patterns, justifying our interpretation of PWS as "plant-water" sensitivity.

We add the results from our investigation into the drivers of PWS in the manuscript in the lines L165:

> The strong link between PWS and $\frac{d\ burned\ area}{d\ VPD}$ prompts the question, what factors affect PWS? Using a random forest model (see Methods), we find that 57% of the variance in PWS is explained by 13 plant and soil hydraulic traits (Fig. S9). The most important drivers of PWS are average saturated soil hydraulic conductivity (25% importance), average saturated volumetric water content (10% importance), and isohydricity—which describes xylem and stomatal regulation (9% importance; Fig. 2). In total, soil and plant traits contribute to 54% and 46% of the explained importance, respectively.

We also discuss the results in the Discussion section L246:

> Although PWS is not a widely recognized plant trait, we show that it is an indicator of whole-plant water relations. It combines several plant and soil hydraulic traits that affect LFMC (Fig. 2). Notably, soil hydraulic traits explain a slightly larger fraction (54% vs. 46%) of the variation in PWS than plant hydraulic traits do. This is to some extent expected; datasets of the explanatory variables have much coarser resolution than that of PWS, and because vegetation traits are more spatially heterogeneous than soil traits, this mismatch in scales will add noise and reduce the explanatory power of the plant hydraulic traits more than that of the soil hydraulic traits. Furthermore, because PWS is determined from LFMC, a property of the vegetation, even a co-variation with soil hydraulic traits merely reflects the way that plant water uptake is affected by soil hydraulics. Thus, we argue that our interpretation of PWS as a metric of the water sensitivity of plants is appropriate despite the influence of soil hydraulic traits, as further discussed in Text S3.

We include an in-depth interpretation of the variable importance in Text S3:

> Plant and soil hydraulic traits both influence PWS. Since PWS is derived from LFMC's sensitivity to lagged climate-derived moisture balance (see Methods), all hydraulic traits

Open Access This file is licensed under a Creative Commons Attribution 4.0 International License, which permits use, sharing, adaptation, distribution and reproduction in any medium or format, as long as you give appropriate credit to the original author(s) and the source, provide a link to the Creative Commons license, and indicate if changes were made. In the cases where the authors are anonymous, such as is the case for the reports of anonymous peer reviewers, author attribution should be to 'Anonymous Referee' followed by a clear attribution to the source work. The images or other third party material in this file are included in the article's Creative Commons license, unless indicated otherwise in a credit line to the material. If material is not included in the article's Creative Commons license and your intended use is not permitted by statutory regulation or exceeds the permitted use, you will need to obtain permission directly from the copyright holder. To view a copy of this license, visit http://creativecommons.org/licenses/by/4.0/.

that affect LFMC are expected to influence PWS too. We broadly categorize all hydraulic traits as soil traits or plant traits (Table S1), but each trait affects plant moisture in different stages of the soil-plant continuum: from root access to soil water, to water transport through the xylem and leaves, and finally to transpiration. Our ability to identify the importance of the hydraulic traits belonging to each of the stages is limited by data quality. Thus, the relative importance of the variables used in the random forest regression is affected by at least three factors. First, accuracy of data on soil hydraulic traits is higher than plant hydraulic traits. Soil hydraulic traits are derived from intensively sampled in-situ measurements and sub-1-km scale data (Montzka *et al.* 2017). By contrast, most of the plant hydraulic traits we use are retrieved from data assimilation of satellite observables (Liu *et al.* 2021), which are sensitive to errors in the plant hydraulic model underlying the data assimilation, remote sensing retrieval algorithm errors, and uncertainties in the data assimilation methodology. The magnitude of these uncertainties is reflected in the considerable magnitude of the estimated uncertainty for these hydraulic traits (Liu et al., 2021). Second, there is a large mismatch in the spatial resolution of the hydraulic traits and PWS. All hydraulic traits except for canopy height (300 m resolution, though derived from a ten-year old dataset) and root depth (1 km resolution) are available only at 25 km resolution, whereas PWS is available at 4 km resolution. The spatial mismatch may have adversely impacted the importance of plant hydraulic traits, since they are known to be extremely heterogenous (Anderegg 2015). By contrast, the effect of this mismatch should be smaller for soil hydraulic traits, which are generally more homogenous (Liu *et al.* 2014). Third, 43% of the variation in PWS is unaccounted for in our analysis. The unexplained variance might be due to other plant hydraulic traits which are not included in our analysis (Trugman *et al.* 2019). All three above-mentioned factors combined with the near-equal influence of soil and plant hydraulic traits on PWS (Fig. 2) suggests PWS represents whole-plant water relations and not just soil-water relations alone.

We also add a section to the Methods section describing the identification of PWS drivers (L436):

V. c. Spatial drivers of PWS

Open Access This file is licensed under a Creative Commons Attribution 4.0 International License, which permits use, sharing, adaptation, distribution and reproduction in any medium or format, as long as you give appropriate credit to the original author(s) and the source, provide a link to the Creative Commons license, and indicate if changes were made. In the cases where the authors are anonymous, such as is the case for the reports of anonymous peer reviewers, author attribution should be to 'Anonymous Referee' followed by a clear attribution to the source work. The images or other third party material in this file are included in the article's Creative Commons license, unless indicated otherwise in a credit line to the material. If material is not included in the article's Creative Commons license and your intended use is not permitted by statutory regulation or exceeds the permitted use, you will need to obtain permission directly from the copyright holder. To view a copy of this license, visit http://creativecommons.org/licenses/by/4.0/.

To investigate what factors influence PWS, we regress the PWS map against eight plant hydraulic traits and five soil hydraulic traits using an ensemble approach. For plant traits, we use canopy height from 2005 from (Simard *et al.* 2011) and several hydraulic traits including xylem capacitance, a stomatal conductance slope parameter which is inversely proportional to marginal water use efficiency (g_1), maximum xylem conductance, the xylem water potential at 50% loss of conductance (ψ_{50}), and a hydraulic functional type derived from K-means clustering of these plant hydraulic traits determined from (41). We also include isohydricity (a stomatal regulation trait) from (Konings & Gentine 2017), and maximum rooting depth from (Fan *et al.* 2017). For soil traits, we use average saturated hydraulic conductivity (K_s), soil porosity from (Montzka *et al.* 2017) and fraction of soil, silt, and clay from (Liu *et al.* 2014) (Table S1). We rescale all traits to 4-km resolution using nearest neighbor interpolation since all traits (except for canopy height and rooting depth) have a coarse resolution of 25 km.

We use the rescaled traits as explanatory variables in a random forest regression to assess the importance of each trait. We ignore all time-varying factors in our analysis and use static maps of all traits to explain the variance in the static map of PWS. We use 3-fold cross-validation to evaluate strength of fit (Fig. S9). To compute variable importance, we measure the average decrease in node impurity due to splitting at each variable and normalize the output (Fig. 2). For the random forest ensembles, we choose a minimum of six samples per terminal node, a minimum of 5 x 10^{-6} reduction in node impurity at each split, a minimum of two samples split per node, bootstrapped samples during node splits, and 50 trees within each forest. We use scikit-learn for the random forest regression (Pedregosa *et al.* 2011).

Finally, we add the Table and Figures presented above to the supplementary information file as Table S1 and Fig. 2 and S9.

Reviewer: The second reason is that I found that the methodology used to demonstrate the relationship between plant water sensitivity and wildfire vulnerability is not straightforward and this raises a number of issues regarding the meaning and interpretation of the results of Figure 1. Surprisingly, I found no specific discussion in the manuscript on why we should expect that the relationship between dBA/DVP is

Open Access This file is licensed under a Creative Commons Attribution 4.0 International License, which permits use, sharing, adaptation, distribution and reproduction in any medium or format, as long as you give appropriate credit to the original author(s) and the source, provide a link to the Creative Commons license, and indicate if changes were made. In the cases where the authors are anonymous, such as is the case for the reports of anonymous peer reviewers, author attribution should be to 'Anonymous Referee' followed by a clear attribution to the source work. The images or other third party material in this file are included in the article's Creative Commons license, unless indicated otherwise in a credit line to the material. If material is not included in the article's Creative Commons license and your intended use is not permitted by statutory regulation or exceeds the permitted use, you will need to obtain permission directly from the copyright holder. To view a copy of this license, visit http://creativecommons.org/licenses/by/4.0/.

higher for higher PWS. Instead, potential explanations for these results are scattered throughout the text, suggesting that this might be explained by the impact of fuel moisture on fire behavior …

Response: We agree that the potential explanation for the patterns observed in Fig. 1 were not available at one place. We thus restructure the discussion section to have the first paragraph only focus on explaining the pattern observed in Fig. 1. The new paragraph now reads (edits in bold) L217:

> **The relationship in Fig. 1A suggests that vegetation regulates the effect of atmospheric aridity on burned area. This supports our hypothesis that in regions where PWS is high (for e.g., vegetation which keep their stomata relatively open, or have only shallow roots in a soil where water infiltrates quickly), burned area increases more rapidly per unit increase in VPD than in low PWS regions. The larger increase in burned area in high PWS regions could be due to the greater decline in LFMC during climate-driven water limitation. Such accelerated decline in LFMC can cause a greater increase in fuel flammability or fire spread (Chuvieco *et al.* 2009), thus resulting in larger increase in burned area. The non-linearity in the PWS-$\frac{d\ burned\ area}{d\ VPD}$ relationship could be due to a threshold-like relationship between LFMC and fuel flammability (Dennison & Moritz 2009; Nolan *et al.* 2016).** Despite field-scale experiments showing the effect of plant traits and LFMC on wildfire ignition and spread (Tumino *et al.* 2019; Resco de Dios 2020; Ma *et al.* 2021; Rodman *et al.* 2021), large-scale studies, especially those of burned area, have tended to ignore the effects of plant physiology, with some exceptions (Abatzoglou *et al.* 2018; Littell *et al.* 2018; Hurteau *et al.* 2019). Although the relationship between inter-annual burned area and climate aridity is strong at ecoregion- and landscape-scales (Abatzoglou & Kolden 2013; Abatzoglou & Williams 2016; Goss *et al.* 2020), the role of vegetation in amplifying or dampening the effect of aridity on wildfires locally is still poorly understood. **This is in part because LFMC is usually modelled primarily based on meteorological conditions (Bradshaw *et al.* 1984; Hardy & Hardy 2007).** Given our empirical evidence that PWS – and thus, soil and plant hydraulic traits **(Fig. 2)** – regulate burned area variability (Fig. 1), we argue that a greater focus on the ecophysiological controls of burned area is needed to fully understand its drivers, both in the western US and elsewhere. Future studies that explicitly account for spatial variations in PWS or

Open Access This file is licensed under a Creative Commons Attribution 4.0 International License, which permits use, sharing, adaptation, distribution and reproduction in any medium or format, as long as you give appropriate credit to the original author(s) and the source, provide a link to the Creative Commons license, and indicate if changes were made. In the cases where the authors are anonymous, such as is the case for the reports of anonymous peer reviewers, author attribution should be to 'Anonymous Referee' followed by a clear attribution to the source work. The images or other third party material in this file are included in the article's Creative Commons license, unless indicated otherwise in a credit line to the material. If material is not included in the article's Creative Commons license and your intended use is not permitted by statutory regulation or exceeds the permitted use, you will need to obtain permission directly from the copyright holder. To view a copy of this license, visit http://creativecommons.org/licenses/by/4.0/.

> other ecophysiological controls may be better equipped for analyzing and forecasting burned area.

Reviewer: … (e.g., L126) although it is not very clear if the authors refer to fuel moisture or Live fuel moisture. …

Response: We mean live fuel moisture content, but we realize that was not obvious. We thus eliminate the use of the term "fuel moisture" from the manuscript. We now refer to the specific fuel moisture only (live or dead). For example, we edit the sentence under question is to (edit in bold) L131:

> Thus, this potential circularity has little effect on the strong correlation in Fig. 1A, which instead is likely driven by **LFMC's** effects on fire flammability and spread.

Reviewer: … Demonstrating the impact of LFMC on fire behavior is beyond the scope of this manuscript as it would require analyses that specifically relate LFMC estimation with proxies of fire intensity (such as fire severity. However, I think it is important to clearly discuss the main hypotheses that could explain the results shown in Figure 1, notably because dBA/dVPD could be driven by other factors than LFMC. DFMC, for instance, is generally thought to be directly linked to VPD as well as an important driver of fire occurrence.

Response: As mentioned above, we now re-organize and add text discussing the main hypothesis. We agree that dBA/dVPD can be affected by factors other than PWS. In the manuscript, we do not claim that PWS is the sole factor responsible for the differential rise in burned area. As described in Fig. 1, PWS explains 71% of the variance in dBA/dVPD. The rest of the variance may be explained by other factors such DFMC, etc. We add this sentence to the Discussion section to highlight the same L286:

> More generally, the overall relationship between PWS and $\frac{d\ burned\ area}{d\ VPD}$ has 29% of unexplained variance (Fig. 1A). Studies quantifying the effect of dead fuel moisture response, vegetation demographic shifts, and fire behavior on $\frac{d\ burned\ area}{d\ VPD}$ may help resolve this ambiguity.

Open Access This file is licensed under a Creative Commons Attribution 4.0 International License, which permits use, sharing, adaptation, distribution and reproduction in any medium or format, as long as you give appropriate credit to the original author(s) and the source, provide a link to the Creative Commons license, and indicate if changes were made. In the cases where the authors are anonymous, such as is the case for the reports of anonymous peer reviewers, author attribution should be to 'Anonymous Referee' followed by a clear attribution to the source work. The images or other third party material in this file are included in the article's Creative Commons license, unless indicated otherwise in a credit line to the material. If material is not included in the article's Creative Commons license and your intended use is not permitted by statutory regulation or exceeds the permitted use, you will need to obtain permission directly from the copyright holder. To view a copy of this license, visit http://creativecommons.org/licenses/by/4.0/.

Reviewer: Besides, the literature that relates live fuel moisture content to BA and fire behavior, is not mentioned. I think It might help discuss these results (e.g. Dennison and Moritz 2009, Nolan et al. 2016, Pimont et al. 2019)

Response: We add the following sentence to the Discussion section L224:

> The non-linearity in the PWS-$\frac{d\ burned\ area}{d\ VPD}$ relationship could be due to a threshold-like relationship between LFMC and fuel flammability (Dennison & Moritz 2009; Nolan *et al.* 2016).

Reviewer: The rest of the paper is less controversial. However, one key result is that human exposure to wildfire risk grew faster in high areas with high plant water sensitivity but this conclusion is not confirmed by analyses of BA trends. For instance, I would have expected to see analyses showing that for a similar VPD increase, burned area increases more in regions where vegetation moisture shows greater sensitivity to water limitation. But this does not appear in the manuscript. Any reason for this?

Response: We do not fully understand the reviewer's suggestion. The reviewer's comment states:

> "For instance, I would have expected to see analyses showing that for a similar VPD increase, burned area increases more in regions where vegetation moisture shows greater sensitivity to water limitation. But this does not appear in the manuscript. Any reason for this?"

But this is exactly what is shown in Fig. 1 of the manuscript. Because the reviewer mentions human exposure, we guess that maybe the reviewer's concern is about the fact that the pattern of disproportionate WUI growth is not confirmed with analysis of BA trends. It is only presented compared to the PWS hazard zones. If this is indeed the concern, we now analyze the WUI percentage growth within each PWS hazard zone and confirm that the region associated with the fastest relative WUI growth also experienced the largest relative increase in burned area per unit rise in VPD (Fig. R4).

Open Access This file is licensed under a Creative Commons Attribution 4.0 International License, which permits use, sharing, adaptation, distribution and reproduction in any medium or format, as long as you give appropriate credit to the original author(s) and the source, provide a link to the Creative Commons license, and indicate if changes were made. In the cases where the authors are anonymous, such as is the case for the reports of anonymous peer reviewers, author attribution should be to 'Anonymous Referee' followed by a clear attribution to the source work. The images or other third party material in this file are included in the article's Creative Commons license, unless indicated otherwise in a credit line to the material. If material is not included in the article's Creative Commons license and your intended use is not permitted by statutory regulation or exceeds the permitted use, you will need to obtain permission directly from the copyright holder. To view a copy of this license, visit http://creativecommons.org/licenses/by/4.0/.

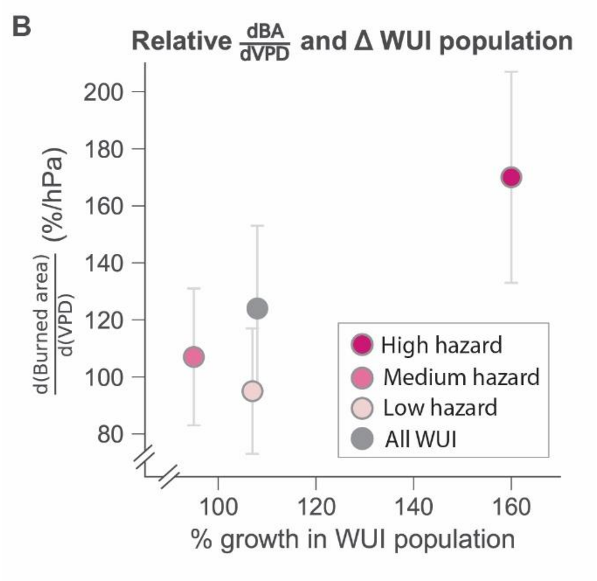

Fig. R4: Rise in burned area (relative to 2001) per unit rise in VPD versus percentage growth in WUI population (relative to 1990) for all three PWS hazard zones. PWS hazard zones are defined in Fig. 1 of the manuscript.

Originally, this figure was not included in the manuscript because it does not contain new information beyond what is already presented in Figs. 1 and 3 of the original manuscript. Fig. 1 shows that dBA/dVPD is linked to PWS. And Fig. 3 shows that % WUI growth has been the highest in high PWS regions. Figure R4 above logically follows. However, we realize that other readers might also have this concern. We thus replace the original B sub-panel (which only showed increase in WUI) with the new Fig. R4. The new figure can be found as Fig. 4B in the manuscript.

Open Access This file is licensed under a Creative Commons Attribution 4.0 International License, which permits use, sharing, adaptation, distribution and reproduction in any medium or format, as long as you give appropriate credit to the original author(s) and the source, provide a link to the Creative Commons license, and indicate if changes were made. In the cases where the authors are anonymous, such as is the case for the reports of anonymous peer reviewers, author attribution should be to 'Anonymous Referee' followed by a clear attribution to the source work. The images or other third party material in this file are included in the article's Creative Commons license, unless indicated otherwise in a credit line to the material. If material is not included in the article's Creative Commons license and your intended use is not permitted by statutory regulation or exceeds the permitted use, you will need to obtain permission directly from the copyright holder. To view a copy of this license, visit http://creativecommons.org/licenses/by/4.0/.

References included in response to all reviewers and in quoted text:

Abatzoglou, J.T. & Kolden, C.A. (2013). Relationships between climate and macroscale area burned in the western United States. *Int. J. Wildl. Fire*, 22, 1003.

Abatzoglou, J.T. & Williams, A.P. (2016). Impact of anthropogenic climate change on wildfire across western US forests. *Proc. Natl. Acad. Sci.*, 113, 11770–11775.

Abatzoglou, J.T., Williams, A.P., Boschetti, L., Zubkova, M. & Kolden, C.A. (2018). Global patterns of interannual climate–fire relationships. *Glob. Chang. Biol.*, 24, 5164–5175.

Anderegg, W.R.L. (2015). Spatial and temporal variation in plant hydraulic traits and their relevance for climate change impacts on vegetation. *New Phytol.*

Arino, O., Perez, J.R., Kalogirou, V., Defourny, P. & Achard, F. (2010). Globcover 2009. In: *ESA Living Planet Symposium*. pp. 1–3.

Balch, J.K., Bradley, B.A., Abatzoglou, J.T., Chelsea Nagy, R., Fusco, E.J. & Mahood, A.L. (2017). Human-started wildfires expand the fire niche across the United States. *Proc. Natl. Acad. Sci. U. S. A.*, 114, 2946–2951.

Bradshaw, L.S., Deeming, J.E., Burgan, R.E. & Cohen, J.D. (1984). *The 1978 National Fire-Danger Rating System: Technical Documentation. General Technical Report INT-169*.

Chuvieco, E., González, I., Verdú, F., Aguado, I. & Yebra, M. (2009). Prediction of fire occurrence from live fuel moisture content measurements in a Mediterranean ecosystem. *Int. J. Wildl. Fire*, 18, 430.

Dennison, P.E. & Moritz, M.A. (2009). Critical live fuel moisture in chaparral ecosystems: A threshold for fire activity and its relationship to antecedent precipitation. *Int. J. Wildl. Fire*, 18, 1021–1027.

Fan, Y., Miguez-Macho, G., Jobbágy, E.G., Jackson, R.B. & Otero-Casal, C. (2017). Hydrologic regulation of plant rooting depth. *Proc. Natl. Acad. Sci. U. S. A.*, 114, 10572–10577.

Open Access This file is licensed under a Creative Commons Attribution 4.0 International License, which permits use, sharing, adaptation, distribution and reproduction in any medium or format, as long as you give appropriate credit to the original author(s) and the source, provide a link to the Creative Commons license, and indicate if changes were made. In the cases where the authors are anonymous, such as is the case for the reports of anonymous peer reviewers, author attribution should be to 'Anonymous Referee' followed by a clear attribution to the source work. The images or other third party material in this file are included in the article's Creative Commons license, unless indicated otherwise in a credit line to the material. If material is not included in the article's Creative Commons license and your intended use is not permitted by statutory regulation or exceeds the permitted use, you will need to obtain permission directly from the copyright holder. To view a copy of this license, visit http://creativecommons.org/licenses/by/4.0/.

Forkel, M., Andela, N., P Harrison, S., Lasslop, G., Van Marle, M., Chuvieco, E., *et al.* (2019). Emergent relationships with respect to burned area in global satellite observations and fire-enabled vegetation models. *Biogeosciences*, 16, 57–76.

García, M., Chuvieco, E., Nieto, H. & Aguado, I. (2008). Combining AVHRR and meteorological data for estimating live fuel moisture content. *Remote Sens. Environ.*, 112, 3618–3627.

Goss, M., Swain, D.L., Abatzoglou, J.T., Sarhadi, A., Kolden, C.A., Williams, A.P., *et al.* (2020). Climate change is increasing the likelihood of extreme autumn wildfire conditions across California. *Environ. Res. Lett.*, 15.

Hantson, S., Arneth, A., Harrison, S.P., Kelley, D.I., Colin Prentice, I., Rabin, S.S., *et al.* (2016). The status and challenge of global fire modelling. *Biogeosciences*, 13, 3359–3375.

Hardy, C.C. & Hardy, C.E. (2007). Fire danger rating in the United States of America: an evolution since 1916. *Int. J. Wildl. Fire*, 16, 217.

Homer, C., Dewitz, J.A., Yang, L., Jin, S., Danielson, P., Xian, G., *et al.* (2011). Completion of the 2006 National Land Cover Database for the conterminous United States. *Photogramm. Eng. Remote Sensing*, 77, 858–566.

Homer, C. & Fry, J. (2012). The National Land Cover Database. *US Geol. Surv. Fact Sheet*, 1–4.

Hurteau, M.D., Liang, S., Westerling, A.L.R. & Wiedinmyer, C. (2019). Vegetation-fire feedback reduces projected area burned under climate change. *Sci. Rep.*, 9, 1–6.

Keeley, J. & Syphard, A. (2016). Climate Change and Future Fire Regimes: Examples from California. *Geosciences*, 6, 37.

Knapp, P.A. (1998). Spatio-temporal patterns of large grassland fires in the Intermountain West, U.S.A. *Glob. Ecol. Biogeogr. Lett.*, 7, 259–272.

Konings, A.G. & Gentine, P. (2017). Global variations in ecosystem-scale isohydricity. *Glob. Chang. Biol.*, 23, 891–905.

Littell, J.S., McKenzie, D., Wan, H.Y. & Cushman, S.A. (2018). Climate Change and Future Wildfire in the Western United States: An Ecological Approach to Nonstationarity. *Earth's Futur.*, 6, 1097–1111.

Open Access This file is licensed under a Creative Commons Attribution 4.0 International License, which permits use, sharing, adaptation, distribution and reproduction in any medium or format, as long as you give appropriate credit to the original author(s) and the source, provide a link to the Creative Commons license, and indicate if changes were made. In the cases where the authors are anonymous, such as is the case for the reports of anonymous peer reviewers, author attribution should be to 'Anonymous Referee' followed by a clear attribution to the source work. The images or other third party material in this file are included in the article's Creative Commons license, unless indicated otherwise in a credit line to the material. If material is not included in the article's Creative Commons license and your intended use is not permitted by statutory regulation or exceeds the permitted use, you will need to obtain permission directly from the copyright holder. To view a copy of this license, visit http://creativecommons.org/licenses/by/4.0/.

Liu, S., Wei, Y., Post, W.M., Cook, R.B., Schaefer, K. & Thorton, M.M. (2014). NACP MsTMIP: Unified North American Soil Map.

Liu, Y., Holtzman, N.M. & Konings, A.G. (2021). Global ecosystem-scale plant hydraulic traits retrieved using model-data fusion. *Hydrol. Earth Syst. Sci.*, 25, 2399–2417.

Ma, W., Zhai, L., Pivovaroff, A., Shuman, J., Buotte, P., Ding, J., *et al.* (2021). Assessing climate change impacts on live fuel moisture and wildfire risk using a hydrodynamic vegetation model. *Biogeosciences*, 18, 4005–4020.

McDowell, N.G., Phillips, N., Lunch, C., Bond, B.J. & Ryan, M.G. (2002). An investigation of hydraulic limitation and compensation in large, old Douglas-fir trees. *Tree Physiol.*, 22, 763–774.

Medlyn, B.E., Duursma, R.A., Eamus, D., Ellsworth, D.S., Prentice, I.C., Barton, C.V.M., *et al.* (2011). Reconciling the optimal and empirical approaches to modelling stomatal conductance. *Glob. Chang. Biol.*, 17, 2134–2144.

Mietkiewicz, N., Balch, J.K., Schoennagel, T., Leyk, S., St. Denis, L.A. & Bradley, B.A. (2020). In the line of fire: Consequences of human-ignited wildfires to homes in the U.S. (1992–2015). *Fire*, 3, 1–20.

Montzka, C., Herbst, M., Weihermüller, L., Verhoef, A. & Vereecken, H. (2017). A global data set of soil hydraulic properties and sub-grid variability of soil water retention and hydraulic conductivity curves. *Earth Syst. Sci. Data*, 9, 529–543.

Nolan, R.H., Boer, M.M., Resco De Dios, V., Caccamo, G. & Bradstock, R.A. (2016). Large-scale, dynamic transformations in fuel moisture drive wildfire activity across southeastern Australia. *Geophys. Res. Lett.*, 43, 4229–4238.

Novick, K., Oren, R., Stoy, P., Juang, J.Y., Siqueira, M. & Katul, G. (2009). The relationship between reference canopy conductance and simplified hydraulic architecture. *Adv. Water Resour.*, 32, 809–819.

Pedregosa, F., Varoquaux, G., Gramfort, A., Michel, V., Thirion, B., Grisel, O., *et al.* (2011). Scikit-learn: Machine Learning in Python. *J. Mach. Learn. Res.*, 12, 2825–2830.

Open Access This file is licensed under a Creative Commons Attribution 4.0 International License, which permits use, sharing, adaptation, distribution and reproduction in any medium or format, as long as you give appropriate credit to the original author(s) and the source, provide a link to the Creative Commons license, and indicate if changes were made. In the cases where the authors are anonymous, such as is the case for the reports of anonymous peer reviewers, author attribution should be to 'Anonymous Referee' followed by a clear attribution to the source work. The images or other third party material in this file are included in the article's Creative Commons license, unless indicated otherwise in a credit line to the material. If material is not included in the article's Creative Commons license and your intended use is not permitted by statutory regulation or exceeds the permitted use, you will need to obtain permission directly from the copyright holder. To view a copy of this license, visit http://creativecommons.org/licenses/by/4.0/.

Rabin, S.S., Melton, J.R., Lasslop, G., Bachelet, D., Forrest, M., Hantson, S., *et al.* (2017). The Fire Modeling Intercomparison Project (FireMIP), phase 1: Experimental and analytical protocols with detailed model descriptions. *Geosci. Model Dev.*, 10, 1175–1197.

Resco de Dios, V. (2020). *Plant-Fire Interactions*. Springer International Publishing.

Rodman, K.C., Veblen, T.T., Andrus, R.A., Enright, N.J., Fontaine, J.B., Gonzalez, A.D., *et al.* (2021). A trait-based approach to assessing resistance and resilience to wildfire in two iconic North American conifers. *J. Ecol.*, 109, 313–326.

Scarff, F.R., Lenz, T., Richards, A.E., Zanne, A.E., Wright, I.J. & Westoby, M. (2021). Effects of plant hydraulic traits on the flammability of live fine canopy fuels. *Funct. Ecol.*, 1–12.

Simard, M., Pinto, N., Fisher, J.B. & Baccini, A. (2011). Mapping forest canopy height globally with spaceborne lidar. *J. Geophys. Res. Biogeosciences*, 116, 1–12.

Syphard, A.D., Keeley, J.E., Pfaff, A.H. & Ferschweiler, K. (2017). Human presence diminishes the importance of climate in driving fire activity across the United States. *Proc. Natl. Acad. Sci. U. S. A.*, 114, 13750–13755.

Trugman, A.T., Anderegg, L.D.L., Sperry, J.S., Wang, Y., Venturas, M. & Anderegg, W.R.L. (2019). Leveraging plant hydraulics to yield predictive and dynamic plant leaf allocation in vegetation models with climate change. *Glob. Chang. Biol.*, 25, 4008–4021.

Tumino, B.J., Duff, T.J., Goodger, J.Q.D. & Cawson, J.G. (2019). Plant traits linked to field-scale flammability metrics in prescribed burns in Eucalyptus forest. *PLoS One*, 14, e0221403.

United States Forest Service. (2018). *National Fuel Moisture Database*. *https://www.wfas.net/nfmd/public/index.php*. Available at: https://www.wfas.net/nfmd/public/index.php. Last accessed 24 December 2018.

Verhoef, A. & Egea, G. (2014). Modeling plant transpiration under limited soil water: Comparison of different plant and soil hydraulic parameterizations and preliminary implications for their use in land surface models. *Agric. For. Meteorol.*, 191, 22–32.

Yebra, M., Aguado, I., García, M., Nieto, H., Chuvieco, E. & Salas, J. (2007). Fuel moisture estimation for fire ignition mapping. *Wildfire2007*, 1–12.

Open Access This file is licensed under a Creative Commons Attribution 4.0 International License, which permits use, sharing, adaptation, distribution and reproduction in any medium or format, as long as you give appropriate credit to the original author(s) and the source, provide a link to the Creative Commons license, and indicate if changes were made. In the cases where the authors are anonymous, such as is the case for the reports of anonymous peer reviewers, author attribution should be to 'Anonymous Referee' followed by a clear attribution to the source work. The images or other third party material in this file are included in the article's Creative Commons license, unless indicated otherwise in a credit line to the material. If material is not included in the article's Creative Commons license and your intended use is not permitted by statutory regulation or exceeds the permitted use, you will need to obtain permission directly from the copyright holder. To view a copy of this license, visit http://creativecommons.org/licenses/by/4.0/.

Decision Letter, first revision:

2nd December 2021

Dear Dr. Rao,

Thank you for submitting your revised manuscript "Plant-Water Sensitivity Regulates Wildfire Vulnerability" (NATECOLEVOL-210814404A). It has now been seen again by the original reviewers and their comments are below. The reviewers find that the paper has improved in revision, and therefore we'll be happy in principle to publish it in Nature Ecology & Evolution, pending minor revisions to satisfy the reviewers' final requests and to comply with our editorial and formatting guidelines.

If the current version of your manuscript is in a PDF format, please email us a copy of the file in an editable format (Microsoft Word or LaTex)-- we can not proceed with PDFs at this stage.

We are now performing detailed checks on your paper and will send you a checklist detailing our editorial and formatting requirements in about a week. Please do not upload the final materials and make any revisions until you receive this additional information from us.

Thank you again for your interest in Nature Ecology & Evolution. Please do not hesitate to contact me if you have any questions.

[REDACTED]

Reviewer #1 (Remarks to the Author):

The authors fully addressed my concerns.

Reviewer #2 (Remarks to the Author):

It is my second review of the manuscript entitled “Plant-Water Sensitivity Regulates Wildfire Vulnerability” by Rao et al. . I read with great interest the new version of the manuscript and the responses by the authors to the reviewer’s comments. The authors did a great job revising the manuscript and addressing the reviewer’s comment. In particular, the authors added robust and relevant analyses (random forests between PWS and soil and hydraulic traits) to assess whether PWS could be associated with specific hydraulic/soil traits. The limits of these analyses are also well described. Furthermore, I appreciated the many changes and text clarification performed by the authors to the manuscript. I just have a couple of comments:

- I have some interrogations regarding the reasons why the average saturated soil hydraulic conductivity (Ks) is the most important factor explaining PWS (about 25%) in the random forest

Open Access This file is licensed under a Creative Commons Attribution 4.0 International License, which permits use, sharing, adaptation, distribution and reproduction in any medium or format, as long as you give appropriate credit to the original author(s) and the source, provide a link to the Creative Commons license, and indicate if changes were made. In the cases where the authors are anonymous, such as is the case for the reports of anonymous peer reviewers, author attribution should be to 'Anonymous Referee' followed by a clear attribution to the source work. The images or other third party material in this file are included in the article’s Creative Commons license, unless indicated otherwise in a credit line to the material. If material is not included in the article’s Creative Commons license and your intended use is not permitted by statutory regulation or exceeds the permitted use, you will need to obtain permission directly from the copyright holder. To view a copy of this license, visit http://creativecommons.org/licenses/by/4.0/.

analyses. In my understanding of plant hydraulics, soil conductivity plays a minor role in plant water dynamics. I, therefore, suspect Ks to be a proxy for other soil characteristics linked to water retention curves.

- I also wondered why the authors removed the citations to some studies discussing how the different plant functional strategies between species or plant functional types might explain LFMC dynamics (references 24-26) in the previous version of the manuscript. As an author of one of these manuscripts, I am uncomfortable recommending these papers for citation. However, since they provide interesting material regarding the link between plant water potential, LFMC and soil/hydraulic traits, it seemed to be relevant to include them.

Our ref: NATECOLEVOL-210814404A

10th December 2021

Dear Dr. Rao,

Thank you for your patience as we've prepared the guidelines for final submission of your Nature Ecology & Evolution manuscript, "Plant-Water Sensitivity Regulates Wildfire Vulnerability" (NATECOLEVOL-210814404A). Please carefully follow the step-by-step instructions provided in the attached file, and add a response in each row of the table to indicate the changes that you have made. Please also check and comment on any additional marked-up edits we have proposed within the text. Ensuring that each point is addressed will help to ensure that your revised manuscript can be swiftly handed over to our production team.

We would like to start working on your revised paper, with all of the requested files and forms, as soon as possible (preferably within two weeks). Please get in contact with us immediately if you anticipate it taking more than two weeks to submit these revised files.

When you upload your final materials, please include a point-by-point response to any remaining reviewer comments.

If you have not done so already, please alert us to any related manuscripts from your group that are under consideration or in press at other journals, or are being written up for submission to other journals (see: https://www.nature.com/nature-research/editorial-policies/plagiarism#policy-on-duplicate-publication for details).

Open Access This file is licensed under a Creative Commons Attribution 4.0 International License, which permits use, sharing, adaptation, distribution and reproduction in any medium or format, as long as you give appropriate credit to the original author(s) and the source, provide a link to the Creative Commons license, and indicate if changes were made. In the cases where the authors are anonymous, such as is the case for the reports of anonymous peer reviewers, author attribution should be to 'Anonymous Referee' followed by a clear attribution to the source work. The images or other third party material in this file are included in the article's Creative Commons license, unless indicated otherwise in a credit line to the material. If material is not included in the article's Creative Commons license and your intended use is not permitted by statutory regulation or exceeds the permitted use, you will need to obtain permission directly from the copyright holder. To view a copy of this license, visit http://creativecommons.org/licenses/by/4.0/.

In recognition of the time and expertise our reviewers provide to Nature Ecology & Evolution's editorial process, we would like to formally acknowledge their contribution to the external peer review of your manuscript entitled "Plant-Water Sensitivity Regulates Wildfire Vulnerability". For those reviewers who give their assent, we will be publishing their names alongside the published article.

Nature Ecology & Evolution offers a Transparent Peer Review option for new original research manuscripts submitted after December 1st, 2019. As part of this initiative, we encourage our authors to support increased transparency into the peer review process by agreeing to have the reviewer comments, author rebuttal letters, and editorial decision letters published as a Supplementary item. When you submit your final files please clearly state in your cover letter whether or not you would like to participate in this initiative. Please note that failure to state your preference will result in delays in accepting your manuscript for publication.

Cover suggestions

As you prepare your final files we encourage you to consider whether you have any images or illustrations that may be appropriate for use on the cover of Nature Ecology & Evolution.

Covers should be both aesthetically appealing and scientifically relevant, and should be supplied at the best quality available. Due to the prominence of these images, we do not generally select images featuring faces, children, text, graphs, schematic drawings, or collages on our covers.

We accept TIFF, JPEG, PNG or PSD file formats (a layered PSD file would be ideal), and the image should be at least 300ppi resolution (preferably 600-1200 ppi), in CMYK colour mode.

If your image is selected, we may also use it on the journal website as a banner image, and may need to make artistic alterations to fit our journal style.

Please submit your suggestions, clearly labeled, along with your final files. We'll be in touch if more information is needed.

Nature Ecology & Evolution has now transitioned to a unified Rights Collection system which will allow our Author Services team to quickly and easily collect the rights and permissions required to publish your work. Approximately 10 days after your paper is formally accepted, you will receive an email in providing you with a link to complete the grant of rights. If your paper is eligible for Open Access, our Author Services team will also be in touch regarding any additional information that may be required to arrange payment for your article.

Please note that <i>Nature Ecology & Evolution</i> is a Transformative Journal (TJ). Authors may publish their research with us through the traditional subscription access route or make their paper immediately open access through payment of an article-processing charge (APC). Authors will not be required to make a final decision about access to their article until it has been accepted. Find out more

Open Access This file is licensed under a Creative Commons Attribution 4.0 International License, which permits use, sharing, adaptation, distribution and reproduction in any medium or format, as long as you give appropriate credit to the original author(s) and the source, provide a link to the Creative Commons license, and indicate if changes were made. In the cases where the authors are anonymous, such as is the case for the reports of anonymous peer reviewers, author attribution should be to 'Anonymous Referee' followed by a clear attribution to the source work. The images or other third party material in this file are included in the article's Creative Commons license, unless indicated otherwise in a credit line to the material. If material is not included in the article's Creative Commons license and your intended use is not permitted by statutory regulation or exceeds the permitted use, you will need to obtain permission directly from the copyright holder. To view a copy of this license, visit http://creativecommons.org/licenses/by/4.0/.

about Transformative Journals

<B>Authors may need to take specific actions to achieve compliance with funder and institutional open access mandates. For submissions from January 2021, if your research is supported by a funder that requires immediate open access (e.g. according to Plan S principles) then you should select the gold OA route, and we will direct you to the compliant route where possible. For authors selecting the subscription publication route our standard licensing terms will need to be accepted, including our self-archiving policies. Those standard licensing terms will supersede any other terms that the author or any third party may assert apply to any version of the manuscript.

Please note that you will not receive your proofs until the publishing agreement has been received through our system.

For information regarding our different publishing models please see our Transformative Journals page. If you have any questions about costs, Open Access requirements, or our legal forms, please contact ASJournals@springernature.com.

Please use the following link for uploading these materials:
[REDACTED]

If you have any further questions, please feel free to contact me.

[REDACTED]

Reviewer #1:
Remarks to the Author:
The authors fully addressed my concerns.

Reviewer #2:
Remarks to the Author:
It is my second review of the manuscript entitled “Plant-Water Sensitivity Regulates Wildfire Vulnerability” by Rao et al. . I read with great interest the new version of the manuscript and the responses by the authors to the reviewer’s comments. The authors did a great job revising the manuscript and addressing the reviewer’s comment. In particular, the authors added robust and

Open Access This file is licensed under a Creative Commons Attribution 4.0 International License, which permits use, sharing, adaptation, distribution and reproduction in any medium or format, as long as you give appropriate credit to the original author(s) and the source, provide a link to the Creative Commons license, and indicate if changes were made. In the cases where the authors are anonymous, such as is the case for the reports of anonymous peer reviewers, author attribution should be to 'Anonymous Referee' followed by a clear attribution to the source work. The images or other third party material in this file are included in the article’s Creative Commons license, unless indicated otherwise in a credit line to the material. If material is not included in the article’s Creative Commons license and your intended use is not permitted by statutory regulation or exceeds the permitted use, you will need to obtain permission directly from the copyright holder. To view a copy of this license, visit http://creativecommons.org/licenses/by/4.0/.

relevant analyses (random forests between PWS and soil and hydraulic traits) to assess whether PWS could be associated with specific hydraulic/soil traits. The limits of these analyses are also well described. Furthermore, I appreciated the many changes and text clarification performed by the authors to the manuscript. I just have a couple of comments:

- I have some interrogations regarding the reasons why the average saturated soil hydraulic conductivity (Ks) is the most important factor explaining PWS (about 25%) in the random forest analyses. In my understanding of plant hydraulics, soil conductivity plays a minor role in plant water dynamics. I, therefore, suspect Ks to be a proxy for other soil characteristics linked to water retention curves.

- I also wondered why the authors removed the citations to some studies discussing how the different plant functional strategies between species or plant functional types might explain LFMC dynamics (references 24-26) in the previous version of the manuscript. As an author of one of these manuscripts, I am uncomfortable recommending these papers for citation. However, since they provide interesting material regarding the link between plant water potential, LFMC and soil/hydraulic traits, it seemed to be relevant to include them.

Author Rebuttal, first revision:

Reviewer #2 (Remarks to the Author):

Reviewer: - I have some interrogations regarding the reasons why the average saturated soil hydraulic conductivity (Ks) is the most important factor explaining PWS (about 25%) in the random forest analyses. In my understanding of plant hydraulics, soil conductivity plays a minor role in plant water dynamics. I, therefore, suspect Ks to be a proxy for other soil characteristics linked to water retention curves.

Response: To check whether the importance of soil hydraulic conductivity is high because it acts as a surrogate to the soil-water retention curves, or whether it has inherit predictive ability, we add a new explanatory variable n to the random forest regression of PWS. The n is a shape parameter in soil water retention curve. It is the exponent in the pedo-transfer function in the van Genuchten model. The map of n was obtained from (Montzka *et al.* 2017). With the additional variable, the random forest regression's variable importance is shown below.

Open Access This file is licensed under a Creative Commons Attribution 4.0 International License, which permits use, sharing, adaptation, distribution and reproduction in any medium or format, as long as you give appropriate credit to the original author(s) and the source, provide a link to the Creative Commons license, and indicate if changes were made. In the cases where the authors are anonymous, such as is the case for the reports of anonymous peer reviewers, author attribution should be to 'Anonymous Referee' followed by a clear attribution to the source work. The images or other third party material in this file are included in the article's Creative Commons license, unless indicated otherwise in a credit line to the material. If material is not included in the article's Creative Commons license and your intended use is not permitted by statutory regulation or exceeds the permitted use, you will need to obtain permission directly from the copyright holder. To view a copy of this license, visit http://creativecommons.org/licenses/by/4.0/.

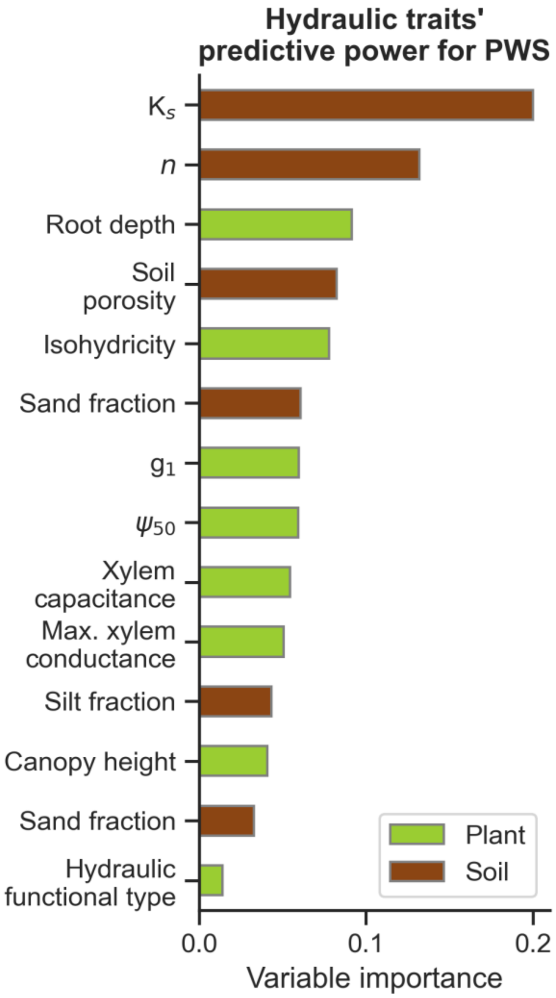

In the new model, the soil hydraulic conductivity at saturation (K_s) still appears as the most important variable, but with less relative importance (20% now as compared to 25% earlier) and *n* is the 2nd most important variable (relative importance = 13%). The marginal reduction in K_s's importance shows that it was likely correlated to *n* and acted as an imperfect surrogate for the soil water retention curve, but since its importance is high (20%) even after adding *n*, it does hold predictive ability by itself, which could be due to the effect of deep soil percolation on root zone water availability for vegetation as noted earlier. We describe the changes in these new lines in the Results section (L123 in changes highlighted version):

Open Access This file is licensed under a Creative Commons Attribution 4.0 International License, which permits use, sharing, adaptation, distribution and reproduction in any medium or format, as long as you give appropriate credit to the original author(s) and the source, provide a link to the Creative Commons license, and indicate if changes were made. In the cases where the authors are anonymous, such as is the case for the reports of anonymous peer reviewers, author attribution should be to 'Anonymous Referee' followed by a clear attribution to the source work. The images or other third party material in this file are included in the article's Creative Commons license, unless indicated otherwise in a credit line to the material. If material is not included in the article's Creative Commons license and your intended use is not permitted by statutory regulation or exceeds the permitted use, you will need to obtain permission directly from the copyright holder. To view a copy of this license, visit http://creativecommons.org/licenses/by/4.0/.

> The most important drivers of PWS are average saturated soil hydraulic conductivity (K_s, 20% importance), the shape of soil water retention curves (*n*, 13% important), and root depth (9% importance; Fig. 2). In total, soil and plant traits contribute to 55% and 45% of the explained importance, respectively.

We also update Fig. 2 to the new version (as shown above). Finally, we update Table S1 to add details about the *n* parameter.

Reviewer: I also wondered why the authors removed the citations to some studies discussing how the different plant functional strategies between species or plant functional types might explain LFMC dynamics (references 24-26) in the previous version of the manuscript. As an author of one of these manuscripts, I am uncomfortable recommending these papers for citation. However, since they provide interesting material regarding the link between plant water potential, LFMC and soil/hydraulic traits, it seemed to be relevant to include them.

Response: We agree that the above-mentioned references provide important insights in the introduction of our manuscript. We had removed the mentioned lines to fit the revised manuscript within the word limit of 3500 words (since changes requested by both authors required several new sentences). We have now inserted the above-mentioned sentences and associated references back into the manuscript at L. 45. Due to this change, we overshoot the word limit by about 15 words. We request the editor to allow the extra words.

References included in response to all reviewers and in quoted text:

Montzka, C., Herbst, M., Weihermüller, L., Verhoef, A. & Vereecken, H. (2017). A global data set of soil hydraulic properties and sub-grid variability of soil water retention and hydraulic conductivity curves. *Earth Syst. Sci. Data*, 9, 529–543.

Open Access This file is licensed under a Creative Commons Attribution 4.0 International License, which permits use, sharing, adaptation, distribution and reproduction in any medium or format, as long as you give appropriate credit to the original author(s) and the source, provide a link to the Creative Commons license, and indicate if changes were made. In the cases where the authors are anonymous, such as is the case for the reports of anonymous peer reviewers, author attribution should be to 'Anonymous Referee' followed by a clear attribution to the source work. The images or other third party material in this file are included in the article's Creative Commons license, unless indicated otherwise in a credit line to the material. If material is not included in the article's Creative Commons license and your intended use is not permitted by statutory regulation or exceeds the permitted use, you will need to obtain permission directly from the copyright holder. To view a copy of this license, visit http://creativecommons.org/licenses/by/4.0/.

Final Decision Letter:

Dear Mr Rao,

We are pleased to inform you that your Article entitled "Plant-Water Sensitivity Regulates Wildfire Vulnerability", has now been accepted for publication in Nature Ecology & Evolution. I enjoyed working with you during this process.

Over the next few weeks, your paper will be copyedited to ensure that it conforms to Nature Ecology and Evolution style. Once your paper is typeset, you will receive an email with a link to choose the appropriate publishing options for your paper and our Author Services team will be in touch regarding any additional information that may be required

After the grant of rights is completed, you will receive a link to your electronic proof via email with a request to make any corrections within 48 hours. If, when you receive your proof, you cannot meet this deadline, please inform us at rjsproduction@springernature.com immediately.

You will not receive your proofs until the publishing agreement has been received through our system

Due to the importance of these deadlines, we ask you please us know now whether you will be difficult to contact over the next month. If this is the case, we ask you provide us with the contact information (email, phone and fax) of someone who will be able to check the proofs on your behalf, and who will be available to address any last-minute problems . Once your paper has been scheduled for online publication, the Nature press office will be in touch to confirm the details.

Acceptance of your manuscript is conditional on all authors' agreement with our publication policies (see www.nature.com/authors/policies/index.html). In particular your manuscript must not be published elsewhere and there must be no announcement of the work to any media outlet until the publication date (the day on which it is uploaded onto our web site).

Please note that <i>Nature Ecology & Evolution</i> is a Transformative Journal (TJ). Authors may publish their research with us through the traditional subscription access route or make their paper immediately open access through payment of an article-processing charge (APC). Authors will not be required to make a final decision about access to their article until it has been accepted. Find out more about Transformative Journals

<B>Authors may need to take specific actions to achieve compliance with funder and institutional open access mandates. For submissions from January 2021, if your research is supported by a funder that requires immediate open access (e.g. according to Plan S

Open Access This file is licensed under a Creative Commons Attribution 4.0 International License, which permits use, sharing, adaptation, distribution and reproduction in any medium or format, as long as you give appropriate credit to the original author(s) and the source, provide a link to the Creative Commons license, and indicate if changes were made. In the cases where the authors are anonymous, such as is the case for the reports of anonymous peer reviewers, author attribution should be to 'Anonymous Referee' followed by a clear attribution to the source work. The images or other third party material in this file are included in the article's Creative Commons license, unless indicated otherwise in a credit line to the material. If material is not included in the article's Creative Commons license and your intended use is not permitted by statutory regulation or exceeds the permitted use, you will need to obtain permission directly from the copyright holder. To view a copy of this license, visit http://creativecommons.org/licenses/by/4.0/.

principles) then you should select the gold OA route, and we will direct you to the compliant route where possible. For authors selecting the subscription publication route our standard licensing terms will need to be accepted, including our self-archiving policies. Those standard licensing terms will supersede any other terms that the author or any third party may assert apply to any version of the manuscript.

In approximately 10 business days you will receive an email with a link to choose the appropriate publishing options for your paper and our Author Services team will be in touch regarding any additional information that may be required.

You will not receive your proofs until the publishing agreement has been received through our system.

If you have any questions about our publishing options, costs, Open Access requirements, or our legal forms, please contact ASJournals@springernature.com

An online order form for reprints of your paper is available at https://www.nature.com/reprints/author-reprints.html. All co-authors, authors' institutions and authors' funding agencies can order reprints using the form appropriate to their geographical region.

We welcome the submission of potential cover material (including a short caption of around 40 words) related to your manuscript; suggestions should be sent to Nature Ecology & Evolution as electronic files (the image should be 300 dpi at 210 x 297 mm in either TIFF or JPEG format). Please note that such pictures should be selected more for their aesthetic appeal than for their scientific content, and that colour images work better than black and white or grayscale images. Please do not try to design a cover with the Nature Ecology & Evolution logo etc., and please do not submit composites of images related to your work. I am sure you will understand that we cannot make any promise as to whether any of your suggestions might be selected for the cover of the journal.

You can now use a single sign-on for all your accounts, view the status of all your manuscript submissions and reviews, access usage statistics for your published articles and download a record of your refereeing activity for the Nature journals.

To assist our authors in disseminating their research to the broader community, our SharedIt initiative provides you with a unique shareable link that will allow anyone (with or without a subscription) to read the published article. Recipients of the link with a subscription will also be able to download and print the PDF.

You can generate the link yourself when you receive your article DOI by entering it here: <a href="http://authors.springernature.com/share">http://authors.springernature.com/share<a>.

Open Access This file is licensed under a Creative Commons Attribution 4.0 International License, which permits use, sharing, adaptation, distribution and reproduction in any medium or format, as long as you give appropriate credit to the original author(s) and the source, provide a link to the Creative Commons license, and indicate if changes were made. In the cases where the authors are anonymous, such as is the case for the reports of anonymous peer reviewers, author attribution should be to 'Anonymous Referee' followed by a clear attribution to the source work. The images or other third party material in this file are included in the article's Creative Commons license, unless indicated otherwise in a credit line to the material. If material is not included in the article's Creative Commons license and your intended use is not permitted by statutory regulation or exceeds the permitted use, you will need to obtain permission directly from the copyright holder. To view a copy of this license, visit http://creativecommons.org/licenses/by/4.0/.
